# Mining and urbanization affect river chemical water quality and macroinvertebrate communities in the upper Selenga River basin, Mongolia (revised version)

**DOI:** 10.1007/s10661-024-13225-6

**Published:** 2024-10-22

**Authors:** Dashdondog Narangarvuu, Tuuguu Enkhdul, Erdenesukh Erdenetsetseg, Enkhbat Enkhrii-Ujin, Khurtsbaatar Irmuunzaya, Gunsmaa Batbayar, Khurelpurev Oyundelger, Rita Sau-Wai Yam, Martin Pfeiffer

**Affiliations:** 1 Department of Biology, National University of Mongolia, Ulaanbaatar, Mongolia; 2 Department of Environmental and Forest Engineering, National University of Mongolia, Ulaanbaatar, Mongolia; 3 College of Environmental Studies, National Dong Hwa University, Shoufeng, Taiwan; 4 Department of Aquatic Ecosystem Analysis and Management (ASAM), Helmholtz Centre for Environmental Research-UFZ, Magdeburg, Germany; 5 Present Address: ADAPT Project, Ulaanbaatar, Mongolia; 6 Department of Botany, Senckenberg Museum of Natural History Görlitz, Görlitz, Germany; 7 Department of Bioenvironmental Systems Engineering, National Taiwan University, Taipei, Taiwan; 8 Department of Biogeography, University of Bayreuth, Universitätsstraße 30, 95447 Bayreuth, Germany

**Keywords:** Bioindicators, Biotic index, Ephemeroptera-Plecoptera-Trichoptera (EPT) complex, Metal pollution, Mongolia

## Abstract

Mongolia is a country with a quickly growing economy mainly based on the mining of gold, copper, coal, and other minerals. Mining, urbanization, and agriculture impact the water quality in the upper Selenga River basin in northern Mongolia, which is the center of the Mongolian economy. Previous measurements of pollution loads were alarming, but restricted to chemical measurements. Here, for the first time, we combine freshwater biomonitoring and laboratory water quality data across a broad gradient of water quality and land use intensity. We track the effects of different types of pollution on aquatic invertebrates and test their use as bioindicators. We collected water samples, environmental parameters, and macroinvertebrates at 36 sampling sites at the rivers of Tuul, Kharaa, and Orkhon and their tributaries Sugnugur, Boroo, Sharyn Gol, Gatsuurt, and Yeröö. PCA of catchment water quality distinguished three groups of pollutants prevalent at the sites, (1) nutrients, (2) salt ions (Cl^−^, Na^+^, Mg^2+^, So_4_^2−^, Ca^2+^) and mining by-products (B, Sr, U, Mo), and (3) (heavy) metals, which often exceeded regulatory standards. We recorded a total of 59 macroinvertebrate taxa belonging to 31 families in seven insect orders plus Amphipoda and Gastropoda. Species diversity declined with higher impact. Five environmental factors structured macroinvertebrate community composition in RDA: elevation of sample location, site total nitrogen, dissolved oxygen, electrical conductivity, and water chemistry. We conclude that macroinvertebrate communities are an appropriate and inexpensive tool for monitoring water quality in Mongolia and suggest government action to establish a long-term monitoring program.

**Supplementary Information:**

The online version contains supplementary material available at 10.1007/s10661-024-13225-6.

## Introduction

River water quality is under serious global threat from the increasing demands of the world’s growing population, rapid economic development, and the effects of climate change (Büttner et al., 2020; IPCC, 2022). In many river basins, especially in the developing world, urbanization, mining, or intensive agriculture has led to extensive sedimentation from river bed erosion and increased surface runoff, metal pollution, and eutrophication from the surrounding landscape or settlements (Fan et al., 2022; Narangarvuu et al., 2014b).

In Mongolia, urbanization and economic development have already led to extensive alteration of riverine environments (Batbayar et al., 2015, 2019). Various types of water pollution (i.e., domestic, industrial, and agricultural activities) affect the quality of river water, hamper the survival of aquatic biota, and even cause the extinction of local populations, posing a potential threat to human health (Batbayar et al., 2017; Narangarvuu et al., 2014b).

The mining sector is a major contributor to the national economy, accounting for 24% of the country’s GDP, 67% of total industrial output, and 93% of its exports in 2020 (Mongolia EITI, 2022). Gold, copper, and coal are the most important export-related minerals in Mongolia. The environmental impact of mining to the river ecosystem includes riverbank erosion, loss of biodiversity, and contamination of groundwater and surface water by anthropogenic chemicals and geogenic minerals (Batsaikhan et al., 2017; Battogtokh et al., 2014; Pfeiffer et al., 2015). In Mongolia, open placer gold mining by illegal miners (“ninja”) is widespread and causes further negative impacts, such as the use of mercury and cyanide for panning the gold (Byambaa & Todo, 2011; Oyuntsetseg et al., 2012).

Aquatic insects are important inhabitants of river ecosystem due to their high abundance, taxonomic diversity, and importance in many ecological processes. They play diverse roles in nutrient processing and energy flow in river food webs (Narangarvuu et al., 2014a, b). Together with other aquatic invertebrates, they have been shown to regulate the structure and functions (e.g., productivity, energy transfer, and nutrient cycling) of river ecosystems. Therefore, understanding the structural and functional responses of aquatic invertebrate communities along anthropogenic gradients is key to assessing ecosystem response and developing appropriate management strategies at catchment and regional scales (Berger et al., 2017). Aquatic macroinvertebrates are often used as indicators for assessing human impacts on river ecosystems. There are a number of reasons for this, including their diverse life histories, their wide distribution and relative ease of taxonomic identification, their multiple functions in water and sediment, and their varying tolerance to pollution and ecological fluctuations (Feio et al., 2007; Pfeiffer et al., 2021; Rico-Sánchez et al., 2022). Effects of water pollution and sedimentation on their community structure have been well-documented in temperate regions (Assefa et al., 2023; Goncharov et al., 2020; Narangarvuu et al., 2014b), including Mongolia (Avlyush et al., 2018; Morse et al., 2007).

In the southern part of the upper Selenga River basin in North East Mongolia, Tuul, Kharaa, and Orkhon Rivers flow through the three major cities of Mongolia (Ulaanbaatar, Darkhan, and Erdenet), which are key agricultural, industrial, and mining areas (in particular for gold and copper). This not only increases water consumption, but also increases the chance of water contamination. Although the Selenga’s tributaries flow largely through rural areas, there is point and non-point pollution from industrial mining and artisanal gold panning operations, from intensive livestock and scattered crop farming, and from municipal discharges. Treated and untreated sewage are contributing to pollution, which has the potential to harm a large number of people (Chalov et al., 2013).

A growing body of literature has studied the impact of urbanization, mining, and intensified livestock breeding on the water quality at the upper Selenga Basin in northern Mongolia (e.g., Batbayar et al., 2015, 2017; Byambaa & Todo, 2011; Hofmann et al., 2015; Karthe et al., 2019; Kasimov et al., 2017; Krätz et al., 2010; Zandaryaa et al., 2013). Water chemistry (e.g., Thorslund et al., 2012) or sediment load (e.g., Jarsjo et al., 2017) has been investigated, while only a few articles considered the impact of water pollution on aquatic organisms (Avlyush, 2011; Kaus et al., 2017). However, recently, researchers investigated the impact of sediment load (Schäffer et al., 2019) and land use change and grazing (Yadamsuren et al., 2020, 2022) on macroinvertebrate communities in the upper Selenga. Pfeiffer et al. (2021) studied the response of macroinvertebrates to water chemistry, but they focused on an upper tributary of the Kharaa River, with limited anthropogenic influences. Thus, up to now, no detailed study has been attempted to quantify the responses of macroinvertebrate communities along the pollution gradients in Mongolia.

Considering pollution from mining, agriculture, and urbanization, we propose that aquatic macroinvertebrates can serve as a useful tool for tracking the effects of different types of pollution on the environment of northern Mongolia. We expect to identify bioindicator taxa that respond specifically to their aquatic surrounding and are bound to their ecological niches. At the same time, invertebrate communities should differ significantly in their composition depending on the source and impact of pollutants.

Our findings will be useful for designing biomonitoring procedures and river restoration programs and will help to develop innovative approaches to deepen and diversify our knowledge of Mongolian river ecosystems.

## Material and methods

### Study area

**Fig. 1:**
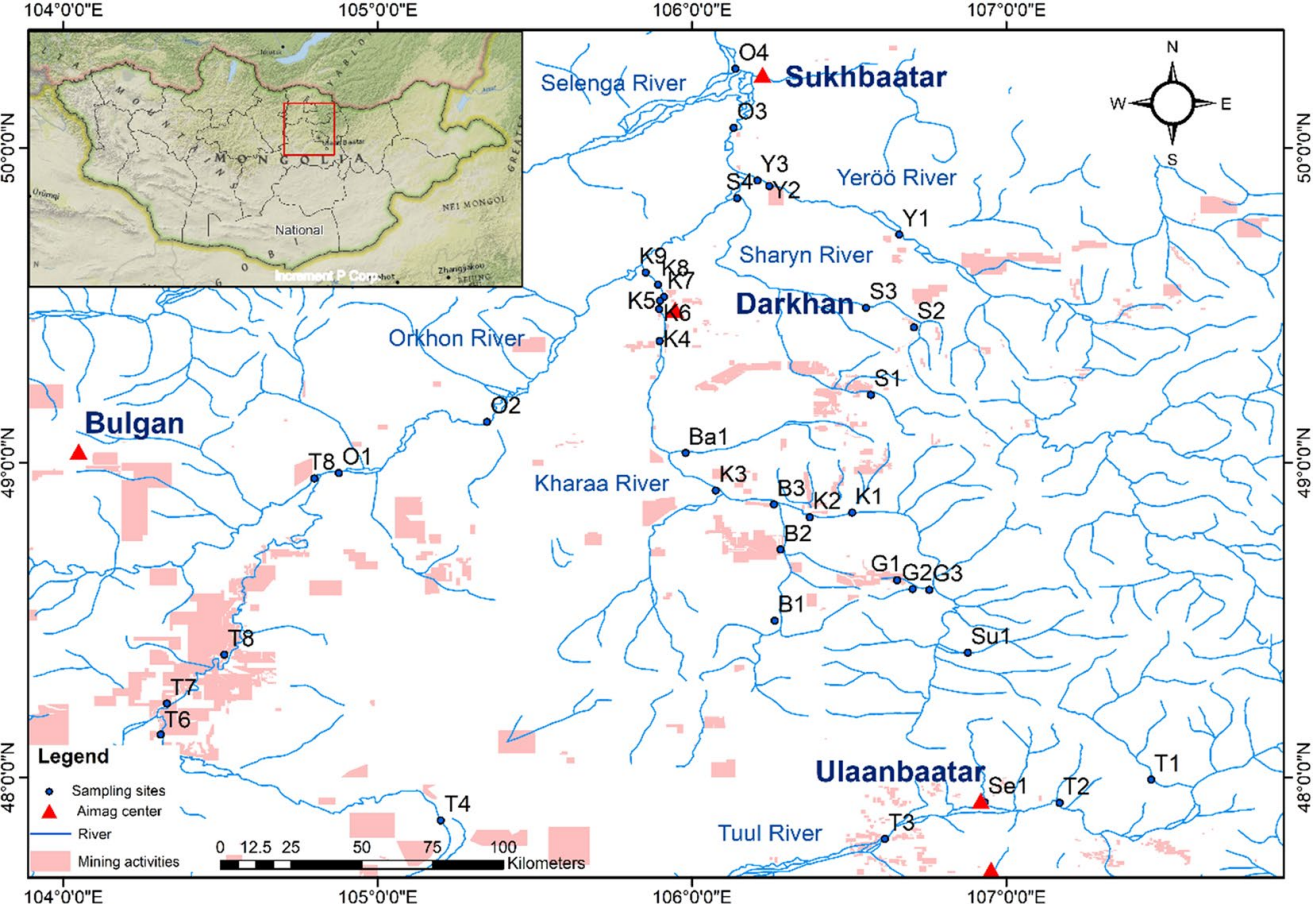
Location of our survey sites in the southern upper Selenga basin in northern Mongolia

The Selenga Basin has a catchment area of 425,245 km^2^, covering 30.6% of Mongolia’s water resources and spanning a variety of ecoregions including high mountains, taiga, forest steppe, and steppe (Bazha et al., 2019; Tumurchudur & Jadambaa, 2012). Our study area in northern Mongolia includes the southern part of the upper Selenga basin, namely the Orkhon, Tuul, and Kharaa catchments. It includes rivers such as Orkhon, Yeröö, Kharaa, Sharyn Gol, Khuiten, Bayangol, Gatsuurt, Boroo, Sugnugur, Tuul, and Selbe, which are affected by agriculture, mining, industry, and settlements. The sampling sites are shown in Fig. 1, and the characteristics of the main rivers are described below.

i. Tuul River. The water quality of the Tuul River is relatively good upstream until Ulaanbaatar (UB), the capital of Mongolia, with a population of about 1.5 million. Here, the small Selbe River joins into the Tuul. Further downstream, the Tuul is heavily polluted by discharges from UB’s central wastewater treatment plant (WWTP), which contain high levels of nutrients, oxygen-depleting substances, and chemicals that can significantly affect water quality (Altansukh & Davaa, 2011). A further 300 river km downstream is the Zaamar gold mining area on the banks of the Tuul River. Previous research has shown that inefficient mining methods have resulted in the loss of floodplain habitat, the discharge of large amounts of suspended solids and high levels of phosphorus into the Tuul River, and potential threats to the habitat of local fish species (Batbayar et al., 2017; Jarsjo et al., 2017; Thorslund et al., 2012).
ii. Orkhon River. The Tuul discharges into the Orkhon River, the main tributary of the Selenga River, which feeds Lake Baikal. The Kharaa, Sharyn, and Yeröö rivers also join it.
iii. Kharaa River. The Kharaa River basin covers an area of 14,534 km^2^ in northern Mongolia (Hofmann & Battogtokh, 2017; Zandaryaa et al., 2013). The tributaries of the Kharaa River originate from several relatively pristine valleys of the Khentii Mountains (Pfeiffer et al., 2021). It flows through an area of intensive agricultural use (including large pastures), several small towns, and gold mining areas in the middle reaches. Along its downstream section, around the city of Darkhan, agricultural areas are occasionally interspersed with larger settlements and some industry. A total of 147,000 people live in the Kharaa River basin, half of them in the city of Darkhan. The Kharaa River basin has been the focus of the MoMo integrated water resource management project, which has conducted more than 150 water-related studies of the basin (Avlyush et al., 2018; Karthe et al., 2015).
iv. Gatsuurt River. The Gatsuurt River flows through the Gatsuurt open pit gold mine into the Balj River, which flows into the Kharaa River. The morphology of the Gatsuurt River has also been altered in the past by the construction of roads, earth dams, alluvial deposits, and a surface water reservoir.
v. Boroo River. The Boroo River crosses the gold mining area and is an important water supply for agriculture. The Boroo Gold Mine is an open pit gold mine located approximately 120 km northwest of the capital city of UB in Selenge province in northern Mongolia (Batbayar et al., 2019). Illegal small-scale mining also occurs in this area. Elemental analyses along the Boroo River indicate that downstream waters are impacted by gold mining in the area (Oyuntsetseg et al., 2012).
vi. Sharyn Gol. Sharyn Gol. The Sharyn Gol is a 120-km-long ephemeral river that flows from the headwaters of the western Khentii Mountains and empties into the Orkhon River. A coal mine operates there, and the whole area is affected by small-scale gold mining (McIntyre et al., 2016).
vii. Yeröö River. The Yeröö River is the northernmost tributary of the Orkhon River. The catchment area is covered by forest and steppe and is characterized by intensive livestock breeding (Hofmann & Battogtokh, 2017).

### Recording environmental parameters

Fieldwork was conducted in 2016 from 24 August to 6 September. At each site (Table 1), we conducted a detailed survey of the river to collect data on water quality (water temperature (°C), pH, dissolved oxygen (mg/L), conductivity (mS/cm), nutrients, and dissolved metal/metalloid concentrations (0.2-µm filtration)). Water sampling and recording of water parameters were repeated three times at each site (upstream, midstream, and downstream). At each sampling site, the required physical–chemical parameters were measured on site. In addition, five different water samples were collected, which were treated differently depending on their subsequent analytical route: filtered (0.2-µm and 0.45-µm cellulose acetate filters, Sartorius Stedim Biotech GmbH, Göttingen, Germany), partially acidified (HNO_3_, distilled), or left in their original state. Chemical analyses were performed at the Central Laboratory for Water Analysis & Chemometrics of the Helmholtz Centre for Environmental Research (UFZ) in Magdeburg, Germany, as described in detail in Pfeiffer et al. (2021), using coupled plasma optical emission spectrometry (ICPeOES, Optima 7300 DV, PerkinElmer) and inductively coupled plasma mass spectrometry (ICP-MS/MS; Agilent 8800, Agilent Technologies, Germany).

**Table 1:** Sampling sites of the study (*n* = 36). Given are the sample name, sampling location, river name, altitude, geographic coordinates, and type of human impact on the surrounding area. The samples are sorted upstream-downstream and according to river confluences (see Fig. 1)

Sample	Sampling location	River	Altitude	Longitude	Latitude	Human impacts
T1	Bosgo bridge	Tuul	1545 m	N47° 59.525′	E107° 27.606′	No impacts RP
T2	Gachuurt Tuul River	Tuul	1339 m	N47° 55.124′	E107° 10.150′	Rural
Se1	Selbe River	Selbe	1300 m	N47° 55.228′	E106° 55.862′	Urbanization
T3	Tuul Biocombinate	Tuul	1224 m	N47° 48.230′	E106° 36.795′	WWTP
T4	Tuul Lun bridge	Tuul	997 m	N47° 51.767′	E105° 12.011′	Rural
T5	Tuul Zaamar	Tuul	954 m	N48° 08.144′	E104° 18.572′	Mining
T6	Tuul Zaamar bridge	Tuul	948 m	N48° 14.049′	E104° 19.736′	Mining
T7	Tuul Zaamar downstream	Tuul	917 m	N48° 23.375′	E104° 30.745′	Mining
T8	Orkhon-Tuul junction	Tuul	780 m	N48° 57.011′	E104° 47.890′	Rural
O1	Orkhon-Tuul after junction	Orkhon	775 m	N48° 58.036′	E104° 52.554′	Rural
O2	Orkhon bridge	Orkhon	721 m	N49° 07.782′	E105° 20.862′	Rural
Su1	Sugnugur River	Sugnugur	1151 m	N48° 23.783′	E106° 52.627′	No impacts
G1	Gatsuurt upstream	Gatsuurt	1139 m	N48° 37.616′	E106° 39.114′	Mining
G2	Gatsuurt midstream	Gatsuurt	1066 m	N48° 35.896′	E106° 42.097′	Mining
G3	Gatsuurt downstream	Gatsuurt	1020 m	N48° 35.744′	E106° 45.270′	Mining
K1	Zuunkharaa	Kharaa	852 m	N48° 49.604′	E106° 22.428′	Rural
K2	Kharaa up Boroo	Kharaa	845 m	N48° 49.591′	E106° 22.437′	Mining
B1	Boroo upstream	Boroo	763 m	N48° 29.877′	E106° 15.79′	Mining
B3	Boroo downstream	Boroo	849 m	N48° 52.037′	E106° 15.640′	Mining
K3	Baruunkharaa	Kharaa	805 m	N48° 54.683′	E106° 04.512	Rural
Ba1	Bayangol downstream	Bayangol	782 m	N49° 01.858′	E105° 58.745′	Agriculture
K4	upstream_Darkhan	Kharaa	697 m	N49° 23.205′	E105° 53.773′	Rural
K5	Darkhan Kharaa bridge	Kharaa	679 m	N49° 29.323′	E105° 53.720′	Urbanization
K6	Darkhan WWTD_upstream	Kharaa	675 m	N49° 30.679′	E105° 53.884′	Urbanization
K7	Darkhan WWTP_down (Island)	Kharaa	675 m	N49° 30.91′	E105° 53.856′	Urbanization/ WWTP
K8	Darkhan_WWTD_downstream	Kharaa	675 m	N49° 31.391′	E105° 53.721′	WWTP
K9	Buren Tolgoi	Kharaa	667 m	N49° 35.559′	E105° 51.641′	Rural
S1	Sharyn upstream	Sharyn	971 m	N49° 12.940′	E106° 34.125′	Rural
S2	Sharyn Khuiten station	Sharyn	928 m	N49° 25.829′	E106° 42.367′	Mining
S3	Sharyn midstream	Sharyn	812 m	N49° 29.546′	E106° 33.224′	Mining
S4	Sharyn Jimst station	Sharyn	621 m	N49° 50.466′	E106° 08.592′	Agriculture
Y1	Yeröö upstream	Yeröö	670 m	N49° 43.517′	E106° 39.552′	Rural
Y2	Yeröö_mid /SRB project/	Yeröö	638 m	N49° 52.774′	E106° 14.725′	Rural
Y3	Yeröö downstream	Yeröö	618 m	N49° 53.835′	E106° 12.483′	Rural
O3	Orkhon Shaamar	Orkhon	619 m	N50° 03.892′	E106° 07.914′	Rural
O4	Orkhon Selenge junction	Orkhon	608 m	N50° 15.160′	E106° 08.272′	Rural

*WWTP* wastewater treatment plant, *RP *reference point

### Macroinvertebrate sampling

To assess the macroinvertebrate fauna, we used quantitative and qualitative sampling methods. Analogous to the water sampling, we collected three replicate samples from different habitats (upstream, midstream, downstream) using an aquatic kick net (0.25 m × 0.25 m area and 250-µm mesh size). The kick net is used to penetrate the river bed to a maximum depth of 0.3 m and filter out free-floating organisms attached to the substrate by moving the bottom substrate within an area of approximately 1 m^2^ above the flow at an inclination of 45°. Filtered macroinvertebrates were collected on white trays. Samples were preserved in 96% alcohol for further study and stored in a laboratory. All macroinvertebrates were identified to the genus level under the microscope (Techno EMT-1-P stereo microscope 40 × , Leica S9i stereo microscope 55 ×) in the laboratory of the first author at the National University of Mongolia using the latest local and international literature and keys (Bouchard, 2012; Chuluunbat et al., 2022; Dashdorj et al., 1969; McCafferty & Provonsha, 1983; Morse et al., 1994; Tsalolikhin, 1997; Zaika, 2000a, b). Some taxa, such as the Chironomidae, were only identified as subgroups. The identified macroinvertebrates were classified and stored according to the standard. The resulting data were entered into a data matrix and prepared for statistical analysis.

### Data processing

**Fig. 2:**
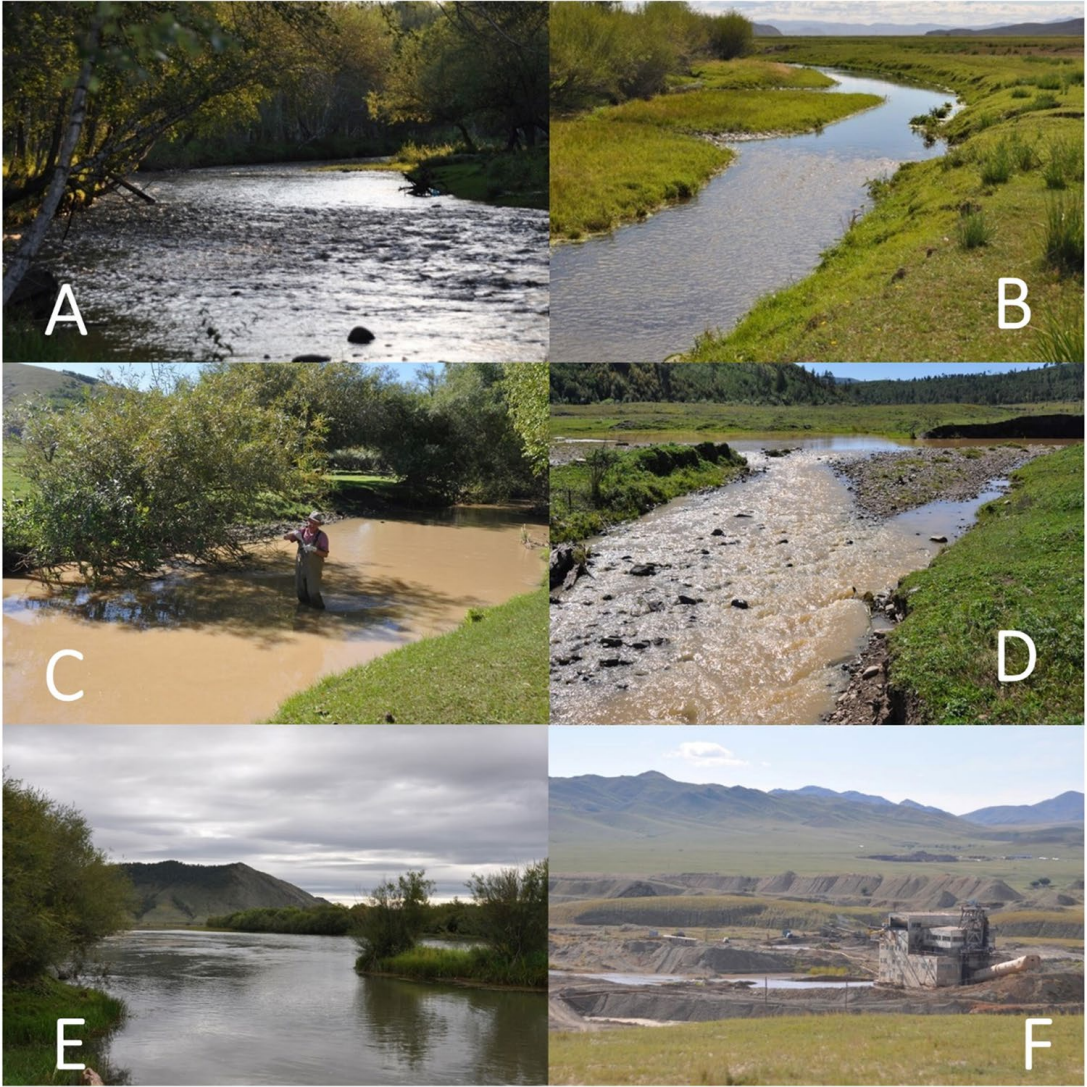
Images of the surveyed sites in northern central Mongolia. **A** Site T1: The upper Tuul River (Tuul B-Bosgo) was used as a comparison site because of the low impact of human activities compared to other sites. **B** Ba1: Bayangol River, with human and livestock impacts. **C** K2: Kharaa River upstream of the confluence with the Boroo River. The whole area is affected by mining. There is also a farm and a sand quarry. **D** G1-3: The Gatsuurt River is affected by mining. **E** Y1, Y2: The Yeröö River is affected by settlements, agriculture, and livestock. Almost half of the Yeröö River basin is covered by forest. **F** T5-T7 The Tuul River around the mining area in Zaamar soum in Tuv Aimag

For each of the sample sites (Figs. 1, 2; Table 1), we collected a set of physicochemical parameters (Online Resource, Table A.1). One site had to be excluded from the analysis, due to missing data (total *n* = 36).

We quantified the macroinvertebrate diversity of each site with R package *vegan* (Oksanen et al., 2018). Using the script of Borcard et al. (2011), we calculated the effective number of species: taxon richness (N0), Shannon diversity, the exponential of Shannon entropy H’ (N1), and the reciprocal of Simpson’s diversity (N2) (see also Hill, 1973).

We assessed the water quality of the rivers using the biotic index according to the formula developed by Hilsenhoff (1987) (Online Resource: Appendix B, Eq: B1, Table B1). Tolerance values for the macroinvertebrate taxa assessed were taken from the literature (Lenat, 1993; Mandaville, 2002).

To calculate the water quality index (WQI) for each sampling site, we took eight chemical measurements (NH_4_^+^-N (mg/L), NO_3_^−^-N (mg/L), NO_2_^−^-N (mg/L), PO_4_^3−^-P (mg/L), Cr (mg/L), Cu (mg/L), Fe (mg/L), Zn (mg/L)). The formula and its evaluation scheme are given in the Online Resource (Appendix B, Eq: B2, Table B2).

We used the Kolmogorov–Smirnov test to validate the normality of the data. Differences between macroinvertebrate community structure and chemical-physical factors at the sampling sites were examined by one-way analysis of variance (ANOVA).

We applied principal component analysis (PCA) to reduce the number of environmental variables and extracted principal components from the main chemical parameters (total Al (mg/L), NH_4_^+^-N (mg/L), total As (µg/L), B (µg/L), Ca^2+^ (mg/L), total Cr (µg/L), Cl (mg/L), Co (µg/L), total Cu (µg/L), total Fe (mg/L), PO_4_^3−^-P (mg/L), K (mg/L), Mg^2+^ (mg/L), Mn (mg/L), Mo (µg/L), Na (mg/L), Ni (µg/L), NO_3_^−^-N (mg/L), NO_2_^−^-N (mg/L), Pb (µg/L), SO_4_ (mg/L), Sr (µg/L), U (µg/L), V (µg/L), Zn (μg/L)).

Macroinvertebrate communities were assessed by redundancy analysis (RDA) with Hellinger transformation of taxon data (Borcard et al., 2011). To evaluate the impact of environmental factors on taxa, we used a set of z-standardized environmental factors including latitude, longitude, altitude, water temperature, WQI, pH, EC, dissolved oxygen (DO), and total bound nitrogen (TNb) of the sites, as well as the first three PCA scores of the water chemistry. We identified the most significant environmental variables using forward selection, but later optimized *R*^2^ and AIC in parsimonious RDA and calculated the variance inflation factor (VIF) to exclude covariates with VIF > 5.5.

We took a *procrustes* rotation in R package *vegan* to compare the structure of PCA and RDA scores and tested the correlation of the matrices with R function *protest* (999 permutations).

## Results

### Water chemistry and environmental factors

**Fig. 3:**
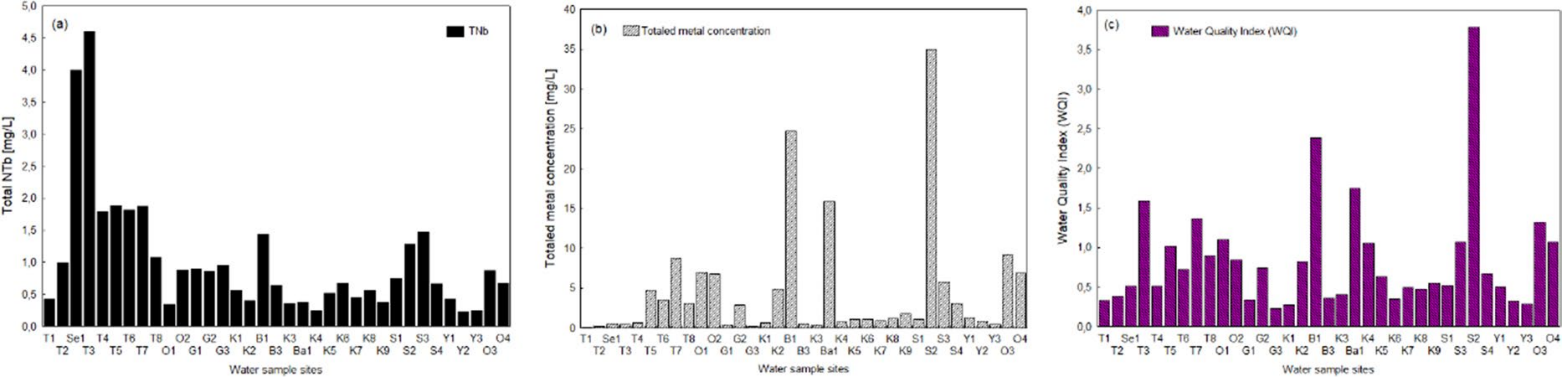
Water pollution in the Selenga River basin. Sampling sites are arranged along the x-axis from upstream to downstream (see Fig. 1, Table 1). **a** Total bound nitrogen. **b** Totaled metal concentrations based on the added concentrations of Al, Co, Cr, Cu, Fe, Mn, Ni, Pb, U, V, and Zn in mg/L. **c** Water quality index, including eight chemical measurements (Cr, Cu, Fe, NH_4_^+^-N, NO_3_^−^-N, NO_2_^−^-N, PO_4_^3−^-P, Zn)

The physicochemical parameters for each of the sampling sites are shown in the Online Resource (Appendix A, Table A.1). Results for TNb, total metal levels, and WQI are given in Fig. 3, while the whole data are presented in the Online Resource (Appendix C, Table C.1).

**Fig. 4:**
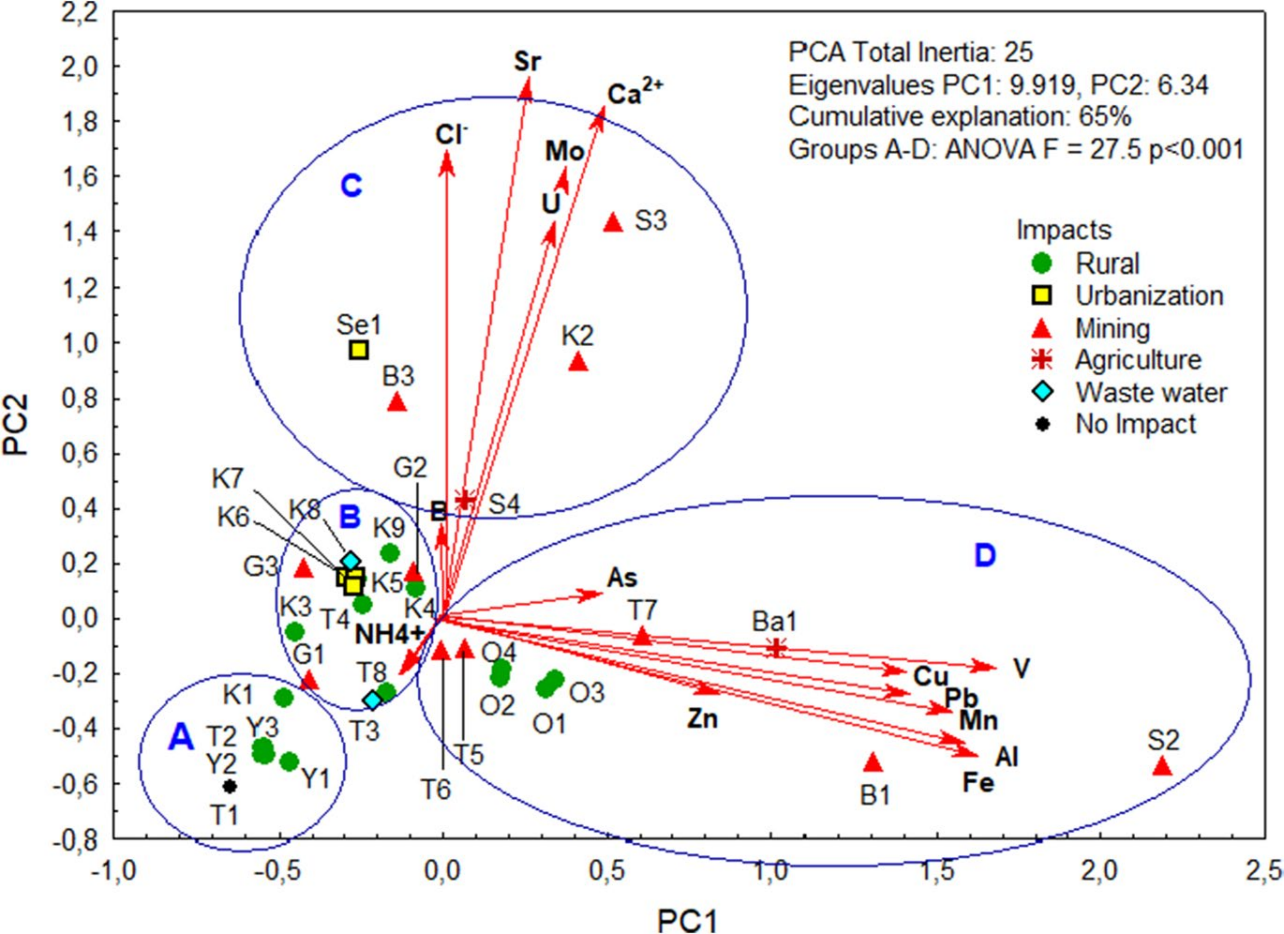
PCA of 25 water chemical parameters at the sampling sites. Shown are the sites marked according to the surrounding land use that affected water quality. Four factors explained 82% of the total variance (see Table D.1 for a full list and their factor loadings). For clarity, we show a two-dimensional subset with only 15 of the chemicals here, mostly metals and semi-metals, but also ammonium (NH_4_^+^), calcium (Ca^2+^), and the pollution indicators chloride (Cl^−^) and boron (B). Site scores (marked with site names, see Table 1) of the samples are marked according to impact group. Four groups of sites (A–D) could be identified across all impacts; their PC1 and PC2 scores differed highly significantly in ANOVA

A PCA explained 82% of the cumulative variance in the data (Online Resource, Table D.1). It divided the sample sites into four groups of different water qualities (Fig. 4), which differed highly significantly in their PCA scores (ANOVA F_(2,6;60)_ = 27.5 *p* = 0.00001): (A) low impact sites, including our no-impact site T1 and other upstream sites such as K1 and Y1; (B) sites with higher levels of nitrogen (NH_4_^+^), but low metal concentrations, e.g., T3 and K8 downstream of WWTPs in UB and Darkhan, but also many sites along the Kharaa River; (C) sites in the positive ordination space of the second PCA axis that were affected by higher concentrations of B, Sr, U, Mo, and ions (Cl^−^, Na^+^, Mg^2+^, SO_4_^2−^, and Ca^2+^), including urban and mining sites; (D) sites in the positive ordination space of the first PCA axis that were affected by higher loads of arsenic and heavy metals. This included the mining sites S2 and B1 and the arable area Ba1, where we found totaled metal concentrations of 35 mg/L, 25 mg/L, and 16 mg/L in the water, respectively, as well as all the downstream rural Orkhon sites affected by the upstream mining in Erdenet and Zaamar, which had totaled metal concentrations between 7 and 9 mg/L.

Rural sites (Fig. 4, green dots) were mainly impacted by livestock herding, which affects water quality mainly through diffuse pollution with nitrogenous manure, resulting in higher ammonium concentrations. On the other hand, point-source nitrogen pollution was measured at T3 at the outlet of the wastewater treatment plant (WWTP, turquoise diamonds) of the capital UB, where NH_4_^+^-N concentrations were seven times higher than the MNS4586 standard (1998) (3.6 mg/L), demonstrating that urbanization is a major driver of nitrogen pollution in water bodies. The high concentration of NH_4_^+^-N is also confirmed by the concentrations of TNb that were highest around the capital city of UB in Tuul River T3 (4.6 mg/L) and Selbe River Se1 (4.0 mg/L). Urbanization (yellow squares) increases general water pollution as indicated by higher concentrations of chloride and boron. Boron (B) compounds enter wastewater mainly via detergent ingredients, but also with fertilizers and from mining tailings (Paliewicz et al., 2014). High B concentrations in our study were found in the mining area (red triangle) at the Gatsuurt River (G2, 99 µg/L; G3, 110 µg/L), in the agricultural area of Ba1 at Bayangol River (153 µg/L) and in the small village of Gachuurt (T2) in the outskirts of UB, below a ger district (198 µg/L). Chloride (Cl^−^) concentrations in our study ranged from a very low 0.8 mg/L in T1 at the upper Tuul to 17.3 mg/L in Sharyn River S3, with high values in the small tributary Selbe River within UB (Se1, 16.2 mg/L) and in the mining areas of Zaamar downstream of the Tuul (T5, T6, T7—10.4 to 11.6 mg/L).

Calcium belongs to the alkaline earth metals and is essential for living organisms. It originates from dissolved rocks (limestone, dolomite) and clays and is a determinant of water hardness. In our samples, the concentrations of Ca^2+^ ions were highly correlated (Spearman correlations) with those of uranium (U) (*r* = 0.67, *p* < 0.001), molybdenum (Mo) (*r* = 0.75, *p* < 0.001), and strontium (Sr) (*r* = 0.88, *p* < 0.001). The highest loads for U (21.1 µg/L) and Mo (9.6 µg/L) were found in the mining area of the Kharaa River upstream of the confluence with the Boroo River (K2), together with high loads for Sr (510 µg/L) and Ca^2+^ (54.3 mg/L). The highest values for Ca^2+^ (54.8 mg/L), Cl^−^ (17.3 mg/L), Mg^2+^ (19.6 mg/L), and Na^+^ (39.3 mg/L), together with high values for Mo (7.4 µg/L), Sr (576 µg/L), and U (14.8 µg/L), were recorded by S3 at Sharyn River, a site 28 km downstream of the mine at Khuiten-Sharyn (S2). The highest concentrations of Sr (785 µg/L) and SO_4_^2−^ (27.1 mg/L) were recorded at Selbe River (Se1) in UB (Online Resource, Table C.1).

Mining activities are responsible for high loads of arsenic and heavy metals in the nearby rivers. Water flow rate is an important parameter and small tributaries can carry higher concentrations. Particularly, high levels of arsenic (As) from mining areas were recorded in the Gatsuurt (G1, 26.0 µg/L; G2, 14.8 µg/L), Kharaa (K2, 12.6 µg/L), and Khuiten-Sharyn (S2, 10.3 µg/L) streams. S2 had also high levels of aluminum (Al) (18.3 mg/L), nickel (Ni) (15.2 µg/L), copper (Cu) (32.1 µg/L), iron (Fe) (13.3 mg/L), and zinc (Zn) (57 µg/L). High metal concentrations were also measured at Boroo Mining (B1), where we found high concentrations of Al (12.8 mg/L), Fe (11.6 mg/L), and lead (Pb) 9.8 µg/L).

In contrast, the Zaamar mining areas on the wide lower reaches of the Tuul River had much lower values, e.g., T7 for As 7.0 µg/L, Cu 9.0 µg/L, Ni 7 µg/L, U 2.7 µg/L, V 16.7 µg/L (see Table C.1). This can be explained by the higher discharge of the main river, possibly pushed by heavy rainfall or the interruption of mining activities. Interestingly, Ba1, an agricultural site, also had high values for Al (8.68 mg/L), U (16.6 µg/L), Ni (9.9 µg/L), and Cu (10.9 µg/L), potentially indicating placer mining in the area.

### Composition of macroinvertebrate communities

A total of 7,368 individuals of macroinvertebrates (59 taxa) were collected from 36 sampling sites in northern Mongolia, belonging into eight orders (Ephemeroptera, Trichoptera, Plecoptera, Amphipoda, Odonata, Hemiptera, Coleoptera, and Diptera), 31 families (Apataniidae, Baetidae, Brachycentridae, Caenidae, Capniidae, Chironomidae, Corixidae, Ephemerellidae, Ephemeriidae, Gammaridae, Georidae, Glossosomatidae, Gomphidae, Gyrinidae, Heptageniidae, Hydrobiosidae, Hydrophilidae, Hydropsychidae, Isonychiidae, Leptophilebiidae, Lestidae, Oligoneuriidae, Perlidae, Perlolidae, Phryganeidae, Psychomyidae, Rhyacophilidae, Simuliidae, Tabanidae, Taeniopterygidae, and Tipulidae), and 55 genera of insects (Online Resource, Table E.1). Ephemeroptera was the most diverse order with 21 genera and followed by Trichoptera (15 genera). According to the ANOVA, Amphipoda (*F* = 2.16, *p* = 0.002), Diptera (*F* = 4.43, *p* = 0.0001), Ephemeroptera (*F* = 4.66, *p* = 0.0001), Hemiptera (*F* = 2.16, *p* = 0.004), Plecoptera (*F* = 2.21, *p* = 0.002), and Trichoptera (*F* = 2.62, *p* = 0.0003) were significantly abundant among the sampling sites. Ephemeroptera was also the most abundant order, occurring at all sites and accounting for 59% of total abundance. Among them, *Baetis* was the most abundant genus, with 37.7% of the total abundance and 65.3% of all individuals of Ephemeroptera. Diptera and Hemiptera were common (13.07% and 15.71%) (Online Resource Fig. E.1). Chironomidae were abundant in almost all sampling sites. Trichoptera made up 7.6% of the total abundance, with *Hydropsyche* as the most dominant genus. The most sensitive group Plecoptera had a relatively low abundance (2.21%) in all sampling sites, and it was only more abundant in the relatively pristine sites, at Sugnugur (Su1) and Sharyn upstream (S1), both headwaters. Here, *Agnetina* was a common genus and was recorded in 14 sampling sites with few individuals.

### Alpha diversity of macroinvertebrate communities

Taxon richness of the invertebrate communities ranged from 4 to 20 taxa per site (Online Resource, Table F.1). We collected a total of 59 taxa (N0) across all sites, giving a Shannon diversity (N1) of 10.54, a Simpson diversity (N2) of 5.23, and a Shannon evenness (E10) of 0.179.

Twenty taxa, representing the highest richness, were recorded in the Kharaa River, both at K3 near Baruunkharaa and at S1 upstream of Sharyn Gol. At G1, the upper Gatsuurt, a tributary of the Kharaa and the Sugnugur River (OW04), we collected 16 taxa. The highest Shannon diversity, 9.76, was calculated for T1, our reference site on the upper Tuul River, representing 15 taxa with a relatively high Shannon evenness of 0.65 (Table F.1). This site was the highest and can be considered free from any harmful human impact.

Only four taxa were collected at the six sites with the lowest taxon richness, B1, K5, O1, T3, T7, and Se1. Shannon diversity N1 of these sites ranged from 1.40 to 3.31, with B1, a mining site upstream of Boroo, having the lowest values for Shannon diversity and evenness (Table F.1).

Species richness and diversity were significantly negatively correlated (Spearman RO correlations, *p* < 0.05) with temperature (N1 =  − 0.35), TNb (N0 =  − 0.45, N1 =  − 0.34), and metal concentrations, viz., Al (N0 =  − 0.38, N1 =  − 0.44), Cu (N0 =  − 0.44, N1 =  − 0.41), and V (N0 =  − 0.40, N1 =  − 0.42), and with higher significance (*p* < 0.01): Cr (N0 =  − 0.45, N1 =  − 0.53), Mn (N0 =  − 0.46, N1 =  − 0.53), Ni (N0 =  − 0.36, N1 =  − 0.47), Pb (N0 =  − 0.44, N1 =  − 0.56). However, the correlations with U and Zn were not significant.

Figure F.1 in the Online Resource shows the distribution of macroinvertebrate diversity at sites grouped by human impact. Although most groups overall did not differ statistically significantly, we can see that taxon diversity was highest under no impact (for N1 and N2: *t*-test, df = 34.1, *t* =  − 2.7 and − 2.8, *p* = 0.01), decreasing progressively with increasing impact and with the lowest values recorded for the urbanization sites (*t*-test, n.s.).

We applied multiple regressions to identify the factors that drove macroinvertebrate diversity in our study (Online Resource, Table G.1). Taxon richness was significantly positively influenced by elevation and longitude of collection sites, as well as by higher values of DO and U, and negatively influenced by higher values for EC (multiple regression adj. *R*^2^ = 0.56, *F*_(5,28)_ = 9.38, *p* < 0.001). Shannon diversity increased with elevation and was negatively affected by higher levels of total nitrogen (multiple regression adj. *R*^2^ = 0.38, *F*_(2,31)_ = 11.13, *p* < 0.001). Simpson diversity at the sites was determined by elevation and longitude of the sites (multiple reg. adj. *R*^2^ = 0.27, *F*_(2,32)_ = 7.21, *p* < 0.01). The strong effect of longitude is due to the general flow direction of the Selenga’s tributaries from southeast to northwest; thus, higher longitude is—like altitude—an indicator of upstream sites.

### Using macroinvertebrate communities as bioindicators

For each of the communities at the sample sites, we calculated the biotic index, which measures the sensitivity of aquatic organisms as an indicator of water quality. A lower index number indicates a lower tolerance to pollution and is therefore evidence of a better water quality. The highest index was found at K8, in Kharaa downstream of the Darkhan WWTP, while the lowest was found at the headwaters of Sugnugur, Su1 (Online Resource Figure H.1). The biotic index was significantly negatively correlated with the effective species number of each site, indicating that higher diversity was associated with lower tolerance (Pearson correlations, *n* = 36; N0, *r* =  − 0.48, *p* < 0.01; N1, *r* =  − 0.59, *p* < 0.001; N2, *r* =  − 0.57, *p* < 0.001).

The index was also negatively correlated with latitude (*r* =  − 0.45, *p* < 0.01) and altitude (*r* =  − 0.033 *p* > 0.05), but positively correlated with temperature (*r* = 0.42, *p* < 0.05), pH (*r* = 0.43, *p* < 0.01), electrical conductivity (EC) (*r* = 0.46, *p* < 0.01), chloride (*r* = 0.52, *p* < 0.01), and water chemistry PC2 (*r* = 0.38, *p* < 0.05). A multiple regression with forward selection of variables explained 51% of the variation and showed that the biotic index was driven by Cl^−^ and NH_4_^+^-N concentrations, while altitude had a negative effect (multiple regression *F*_(3,30)_ = 12.37, *r*^2^ = 0.51, *p* < 0.00002, Online Resource Table G.2).

We searched for bioindicator taxa that should indicate a particular type of land use (Table 1) or one of the four groups of water quality (A–D) identified in the PCA (Fig. 4). Analysis with R function *indicspecies* identified five indicator taxa for no-impact sites of the land use grouping: *Agrypnia* (Phryganeidae), *Anagapetus* and *Glossosoma* (Glossosomatidae), and *Ecdyonurus* (Heptageniidae) were found exclusively at no-impact sites (all stat = 1, *p* = 0.031), while 92% of the abundant *Apatania* (Apataniidae) were recorded there (stat = 0.984, *p* = 0.025). Three indicator taxa could be assigned to the water quality groups of the PCA: *Arcynopteryx* (Perlodidae) (stat = 0.8, *p* = 0.003) and *Heptagenia* (Heptageniidae) (stat = 0.74, *p* = 0.006) indicated group A of water quality sites of the PCA, while midges of the family Orthocladiinae (Chironomidae) indicated group C (stat = 0.83, *p* = 0.022).

We further evaluated the association of specific taxa or taxa groups with water chemistry using Spearman correlation. Odonata were found to be significantly (*p* < 0.05) positively correlated with high concentrations of Mo (*R* = 0.39), U (*R* = 0.34), and Zn (*R* = 0.34). The highly sensitive EPT complex (Ephemeroptera-Plecoptera-Trichoptera) was negatively correlated (*p* < 0.05) in its abundance with concentrations of Cl^−^ (*R* =  − 0.40) and TNb (*R* =  − 0.40). Plecoptera abundance alone was negatively correlated (*p* < 0.01) with Cr (*R* =  − 0.47), Cl^−^ (*R* =  − 0.49), Na^+^ (*R* =  − 0.50), V (*R* =  − 0.35, *p* < 0.05), and others, as well as with PC1 (*R* =  − 0.45, *p* < 0.01). Trichoptera were negatively correlated with Cu (*R* =  − 0.50, *p* < 0.01) and Ni (*R* =  − 0.35, *p* < 0.05).

**Fig. 5:**
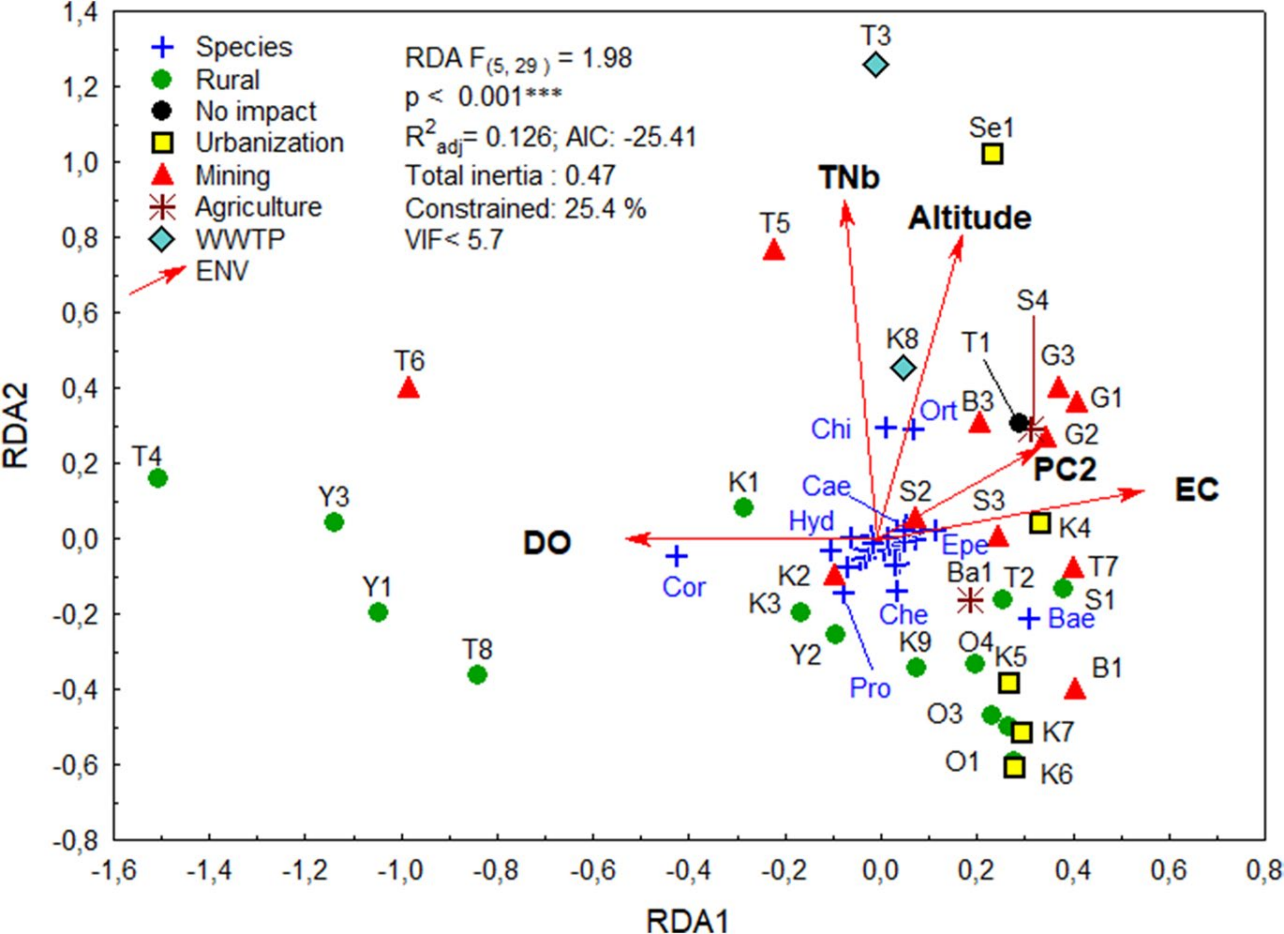
Parsimonious redundancy analysis (RDA) based on the composition of the macroinvertebrate communities (*n* = 36) in our study. Taxon abundances were Hellinger transformed prior to calculation. Formula: tax.h ~ TNb + altitude + DO + EC + PC2; with *p* < 0.001 and variance inflation factors < 5.7. Both RDA axes are significant (*p* < 0.05). Five environmental factors (red arrows) explained 13% of the model variation: Total nitrogen bound (TNb), dissolved oxygen (DO), altitude, electrical conductivity (EC), and the second axis of the PCA (PC2). Site scores of the samples are labelled (see Table 1) and grouped according to the environmental impact at the site. For space limitations, not all points are labelled. Blue crosses mark taxon scores, with some of which are named, including the most abundant macroinvertebrate genera/groups, e.g., Bae, *Baetis*; Cor, Corixidae nymphs; Pro, *Procloeon*; Ort, Orthocladiinae; Chi, Chironomidae; Hyd, *Hydropsyche*; Epe, *Epeorus*; Che, *Cheumatopsyche*; Cae, *Caenis*

We assessed the effects of land use and water chemistry on aquatic invertebrate communities in more detail. The highly significant RDA grouped the macroinvertebrate communities according to the direction of the arrows (Fig. 5): (1) macroinvertebrate assemblages that preferred well-oxygenated water (DO), (2) communities that occurred at sites with high conductivity (EC), (3) communities that tolerated high nutrient concentrations (TNb), or (4) higher concentrations of mining by-products (PC2), and finally (5) communities at higher altitudes.

Along the RDA1 axis, macroinvertebrate communities are sorted along a gradient of dissolved oxygen (DO) and EC, with the rural sites on the left, with high DO and low EC, followed by urban sites in the middle of the gradient, while mining sites are concentrated on the right, indicating high EC in the water. PC2, the second axis of the PCA of water chemistry, also points in this direction, being strongly correlated with the values of Ca^2+^, Cl^−^, Na^+^, SO_4_^2−^, and Sr, which are characteristic of mining effluents.

In contrast, there is a nitrogen gradient along the RDA2 axis, with the highest values at T3, the central WWTP of UB; Se1, the Selbe River, a small tributary of the Tuul that flows directly through a ger district (yurt settlement) of UB; T5, the mining area in Zaamar where many people work without adequate sanitation; and K8, the WWTP of Darkhan, the second largest city in Mongolia. As UB is located on the upper reaches of the Tuul River at an elevation of 1224 m, the highly concentrated wastewater is slowly degraded as it flows downstream to the confluence of the Orkhon and Selenga Rivers (O4) at an elevation of 608 m. The downstream sampling sites on the Orkhon River are all located in the lower right-hand corner of the figure. The mines on the Gatsuurt River (G1–G3) are also very high, between 1139 and 1020 m. The macroinvertebrate communities of the headwater streams (T1, T2, S1) differ only slightly from those of the mountain streams in the mining areas and are close together in the RDA figure. The slightly irritating position of the no-impact site T1 between the mining sites of the Gatsuurt River is due to its high altitude and the low oxygen content of this quietly flowing river (Online Resource, Table A.1). This particular arrangement of sampling sites along contrasting ecological transects may also explain the relatively low explained variance in the RDA (*F*_(5,29)_ = 1.98, *R*^2^_adj_ = 0.13, *p* < 0.001).

The RDA scores were significantly correlated with the PCA scores of the sample sites (*R* = 0.3486, *p* < 0.05), as confirmed by symmetric Procrustes rotation.

## Discussion

Our study is the first to combine freshwater biomonitoring with laboratory water quality data across a wide gradient of water quality and land use intensities in the upper Selenga Basin, Mongolia.

Water quality in this area was highly variable: some pristine sites upstream had excellent water quality, while mining sites often exceeded the limit values for metals and urban sites also failed the standard. At rural sites, we found selective exceedances of MNS limits of unknown origin for some metals (e.g., T2, Cu 11.2 µg/L; K4, Zn 69.3 µg/L). Several sites (B1, Ba1, G1, S2, O3) suffered from a cocktail of metals. Some metals were common contaminants, with 32 of the 36 sites in the Selenga Basin exceeding the MNS4586 standard (1998) for Zn, 20 sites for Al, and 15 sites for Fe (Online Resource Table C.1), confirming the study by Nadmitov et al. (2015). Only three sites (8%) had no limit violations at all.

Such excessive exceedances of government standards for certain substances were also reported by Batbayar et al. (2017). We recorded the third-highest metal pollution at the cropland site Ba1, most likely due to artisanal mining. As river water may be used for irrigation of croplands, authorities should identify potential human health impacts. The highest organic pollution came from the WWTPs in Darkhan and UB, while the distribution of TNb and biotic index showed that the impact of cropland sites (Ba1, S4) on nutrient pollution was limited.

Our results demonstrate that mining, urban development, and agriculture affect water quality and macroinvertebrate communities in different ways. The PCA of the water quality separated a group (A) of unpolluted sites from three other clusters, which were contaminated with various pollutants, namely (B) nutrients, (C) salt components (Cl^−^, Na^+^, Mg^2+^, SO_4_^2−^, Ca^2+^) and other mining by-products (B, Sr, U, Mo), and (D) (heavy) metals.

Similar patterns of pollution have been reported from the Sierra Gorda Biosphere Reserve in the mountains of central Mexico (Rico-Sánchez et al., 2022), which includes undisturbed natural areas affected by industrial and artisanal mining activities, wastewater and agriculture, and is therefore comparable to our study area.

However, in our study, these contaminant groups were not consistent with the land use around the sites; in particular, mining sites were distributed across clusters; and agricultural and rural sites could be heavily contaminated with metals from upstream mining. Overall, this picture corroborated the earlier study of Batbayar et al. (2017), which focused on seasonal trends of water pollution in the Selenga basin and described a similar grouping of their water sampling sites. The high levels of pollution with metals and saline waters indicate that there has been only little improvement in water quality over the last decade (Nadmitov et al., 2015; Zandaryaa et al., 2013). In order to avoid threats to the environment and human health, we strongly recommend closer monitoring of sites with extreme exceedances of legal limits for trace elements, a more precise localization of the sources of pollution, and a reduction of organic contamination in urban river sections.

In our study, RDA results show that not only mining but also urbanization and agriculture contribute to the pollution of surface waters and that these different impacts (DO, EC, PC2, altitude, and TNb) structured macroinvertebrate communities. Comparable results have been reported from tropical Mexico, where macroinvertebrate assemblages were structured by total phosphorous, water and air temperature, and Hg concentration (Rico-Sánchez et al., 2022).

As indicated by the species scores in the RDA figure, macroinvertebrate taxa differed in ecological preference. For example, corixid nymphs dominated site T4, which was characterized by high DO and pH; Chironomidae tolerated high levels of total nitrogen; and the abundant *Baetis* could tolerate high levels of boron and metals. The mayfly genus *Baetis* is one of the largest in Ephemeroptera and includes both sensitive and tolerant species (Buss & Salles, 2007). The family Baetidae is dominant in Mongolian headwater streams (Narangarvuu et al., 2014a). *Baetis vernus*, a common species in Mongolia (Erdenee et al., 2016), is well known to tolerate and accumulate high concentrations of metals such as lead, zinc, and cadmium (Fialkowski et al., 2003). The highest abundance for *Baetis* (569 individuals) was recorded at the agricultural site Ba1, where the totaled metal concentration was 16 mg/L. The stonefly family Perlidae can accumulate metals (Goodyear & McNeill, 1999), and although Plecoptera as a whole was negatively correlated with metals, its genus *Agnetina* (Perlidae) had its highest abundance in S1 and the mining-affected site S2, where extra high concentrations of Al, Fe, Mn, Ni, and Zn were measured with totaled metal concentrations of 35 mg/L. Similarly, Diptera of the genus *Simulium* had their peak abundance at metal-impacted Ba1, and are known to tolerate high total metal contents (Clements et al., 2000). Odonata tolerated high concentrations of Al, as reported from Mexico (Rico-Sánchez et al., 2022). The tolerance values of taxa characterize their resistance to organic water pollution (Table E.1). Caddisflies of the genus *Apatania*, for example, have a low value of 1 and were only recorded in high abundances at our no-impact sites T1 and Su1. They were one of the five significant bioindicators we found for this impact group.

Our study highlights the rich diversity of macroinvertebrates in the waters of the upper Selenga basin, which has already been the focus of other studies (Narangarvuu et al., 2014a; Pfeiffer et al., 2021; Yadamsuren et al., 2022). Diversity was higher at higher elevations and in the eastern (upstream) position and positively influenced by higher values of oxygen and low levels of nitrogen and electrical conductivity. While the lowest diversity was found in a mining area, urbanization and wastewater facilities had a similar effect on diversity. Interestingly, macroarthropod diversity was still high in the upstream Gatsuurt River at G1 and downstream at G3, while it declined midstream at G2 where the highest mining impact occurred, thus pointing to the self-cleaning forces of the watercourse further downstream. For G3, the biotic index showed excellent values and metal concentrations were reduced, probably due to dilution effects and/or sedimentation. In these cases, metal loads were moderate to low, while high pH and higher concentrations of Ca^2+^ and Mg^2+^ from limestone layers in this area buffered the toxic effect of metals by reducing their bioavailability (as in Rico-Sánchez et al., 2022).

## Conclusion

Overall, our results confirm that aquatic macroinvertebrate communities and species respond well to changes in water chemistry, demonstrating their potential as promising biomonitoring tools as known from other regions (Goncharov et al., 2020; Yadamsuren et al., 2022). As hypothesized, we were able to identify indicator species and track changes in invertebrate community composition in relation to pollution patterns.

Environmental monitoring in Mongolia is still mainly conducted by academic institutions, industry, and foreign research projects, while implementation by government agencies is less advanced. However, given the public health implications of metal pollution (Kaus et al., 2017), a more active role for the government is imperative. Long-term invertebrate monitoring of water bodies is a cost-effective method that can form the basis for a comprehensive government monitoring system, which is likely to be essential given the increasing demand for water from mining, agriculture, livestock, and a growing human population.

## Supplementary Information

Below is the link to the electronic supplementary material.

Supplementary file1 (DOCX 395 KB)

## Data Availability

The data that support the findings of this study are listed in the Online Resource; further information is available from the corresponding author upon request.
