# Supplementary material for: Mining and urbanization affect river chemical water quality and macroinvertebrate communities in the upper Selenga River basin, Mongolia (revised version)
Source: Environ Monit Assess. 2024 Oct 22;196(11):1091. doi: 10.1007/s10661-024-13225-6 (PMC11496312; doi:10.1007/s10661-024-13225-6)
Supplement: Supplementary file 1 — Supplementary file1 (DOCX 395 KB) [file 10661_2024_13225_MOESM1_ESM.docx]

Appendix A- Site description

Electronic supplement to Environmental Monitoring and Assessment (corrected 01.01.2024)

Mining and urbanization affect river chemical water quality and macroinvertebrate communities in the upper Selenga River Basin, Mongolia.

*Dashdondog Narangarvuu, Tuuguu Enkhdul, Erdenesukh Erdenetsetseg, Enkhbat Enkhrii-Ujin, Khurtsbaatar Irmuunzaya, Gunsmaa Batbayar, Khurelpurev Oyundelger, Rita Sau-Wai Yam, Martin Pfeiffer**
* Department of Biogeography, University of Bayreuth, Germany; martin.pfeiffer@uni-bayreuth.de

**Table A.1** Means of physicochemical parameters of sample sites. Shown are means of three measurements from slightly different locations, only for Ba1 only one measurement was available. Sites differed in an ANOVA, F and p-values are given in the last lines. For details of sites see Table 1.

| Site | Dissolved oxygen | Temperature | pH | Electric conductivity |
| --- | --- | --- | --- | --- |
| Units | mg/L | ^o^C | - | mS/cm |
| B1 | 8.75±0.24 | 19.7±0.11 | 8.4±0.00 | 0.27±0.003 |
| B3 | 8.76±0.38 | 14.9±0.38 | 7.7±0.01 | 0.43±0.008 |
| Ba1 | 7.17 | 10 | 8.5 | 0.21 |
| G1 | 9.44±0.23 | 15.8±0.40 | 8.6±0.00 | 0.20±0.003 |
| G2 | 9.48±0.29 | 11.9±0.77 | 7.9±0.04 | 0.34±0.01 |
| G3 | 10.4±0.08 | 14.6±0.14 | 8.4±0.01 | 0.33±0.008 |
| K1 | 11.6±0.04 | 16.6±0.17 | 8.4±0.14 | 0.14±0.008 |
| K2 | 9.8±0.17 | 14.6±0.25 | 8.5±0.02 | 0.4±0.005 |
| K3 | 11.8±0.17 | 15.8±0.08 | 8.5±0.01 | 0.21±0.003 |
| K4 | 9.49±0.27 | 17.6±0.18 | 8.2±0.03 | 0.28±0 |
| K5 | 9.02±0.14 | 16.6±0.17 | 8.1±0.02 | 0.27±0.01 |
| K6 | 9.61±0.04 | 20.6±0.25 | 8.4±0.02 | 0.28±0 |
| K7 | 9.59±0.13 | 21.7±0.03 | 8.5±0.01 | 0.27±0.003 |
| K8 | 9.99±0.1 | 22.7±0.35 | 8.6±0.02 | 0.28±0 |
| K9 | 8.15±0.08 | 22.4±0.13 | 8.2±0.02 | 0.3±0 |
| O1 | 8.46±0.07 | 20.9±0.29 | 8.5±0.09 | 0.18±2.00E-17 |
| O2 | 7.52±0.68 | 20.3±0.49 | 8.3±0.01 | 0.18±2.00E-17 |
| O3 | 8.39±0.31 | 19.4±0.29 | 8.0±0.07 | 0.18±2.00E-17 |
| O4 | 6.80±0.24 | 19.3±0.36 | 7.8±0.05 | 0.19±0.01 |
| S1 | 8.47±0.22 | 15.9±0.23 | 8.8±0.02 | 0.24±0.003 |
| S2 | 7.87±0.15 | 16.8±0.51 | 8.1±0.04 | 0.22±0.003 |
| S3 | 8.08±0.13 | 19.0±0.47 | 8.3±0.01 | 0.53±0.003 |
| S4 | 9.37±0.14 | 17.0±0.23 | 8.3±0.08 | 0.34±0.003 |
| Se1 | 9.36±0.11 | 10.8±0.76 | 7.8±0.07 | 0.36±0.01 |
| Su1 | 9.74±0.09 | 10.8±0.44 | 7.1±0.03 | 0.02±0 |
| T1 | 7.78±0.14 | 10.6±0.35 | 7.0±0.15 | 0.02±0 |
| T2 | 8.43±0.11 | 12.9±0.36 | 7.6±0.04 | 0.05±4.90E-18 |
| T3 | 5.25±0.65 | 17.5±0.35 | 7.8±0.08 | 0.16±0.006 |
| T4 | 12.3±0.8 | 18.7±0.2 | 9.4±0.03 | 0.18±2.00E-17 |
| T5 | 9.73±0.32 | 21.5±0.088 | 9.1±0.04 | 0.17±0.003 |
| T6 | 10.4±0.49 | 21.5±0.51 | 9.4±0.06 | 0.18±2.00E-17 |
| T7 | 9.29±0.65 | 21.7±0.58 | 9.2±0.08 | 0.2±0.005 |
| T8 | 8.33±0.28 | 20.6±0.06 | 8.4±0.03 | 0.17±0 |
| Y1 | 8.99±0.16 | 15.4±0.1 | 7.6±0.02 | 0.07±0 |
| Y2 | 9.15±0.61 | 16.6±0.1 | 7.9±0.04 | 0.08±0 |
| Y3 | 9.34±0.02 | 16.2±0.15 | 7.7±0.08 | 0.08±0.003 |
| F value | 15.32 | 92.79 | 31.94 | 319.81 |
| P value | < 0.001 | < 0.001 | < 0.001 | < 0.001 |

Appendix B - Index description

Electronic supplement to Environmental Monitoring and Assessment

Mining and urbanization affect river chemical water quality and macroinvertebrate communities in the upper Selenga River Basin, Mongolia.

*Dashdondog Narangarvuu, Tuuguu Enkhdul, Erdenesukh Erdenetsetseg, Enkhbat Enkhrii-Ujin, Khurtsbaatar Irmuunzaya, Gunsmaa Batbayar, Khurelpurev Oyundelger, Rita Sau-Wai Yam, Martin Pfeiffer**
* Department of Biogeography, University of Bayreuth, Germany; [martin.pfeiffer@uni-bayreuth.de](mailto:martin.pfeiffer@uni-bayreuth.de)

1. **Biotic index (Bi)**

The biotic index developed by Hilsenhoff (1987): $Bi=\frac{\sum x_{i} \times t_{i}}{N}$ **Eq. (B.1)**

Where: $x_{i}$ is the number of individuals of the $i^{th}$ taxa, $t_{i}$ is the type tolerance value of the $i^{th}$ taxa, $N$ = total number of individuals in the sample. Tolerance values for the assessed macroinvertebrate taxa were taken from literature (Lenat 1993; Mandaville 2002).

**Table B.1** Biotic index evaluation list. Given are the numerical value B_i_, the water quality classification group and the degree of organic pollution

| Biotic index | Water quality classification | Degree of organic pollution |
| --- | --- | --- |
| 0.00-3.50 | Excellent | No apparent organic pollution |
| 3.51-4.50 | Very good | Possible slight organic pollution |
| 4.51-5.50 | Good | Some organic pollution |
| 5.51-6.50 | Fair | Fairly significant organic pollution |
| 6.51-7.50 | Fairly poor | Significant organic pollution |
| 7.51-8.50 | Poor | Very significant organic pollution |
| 8.51-10.00 | Very poor | Severe organic pollution |

1. **Water quality index (WQI)**

The water quality index (WQI) was calculated from eight chemical measurements (NH_4_^+^-N (mg/L), NO_3_^-^-N (mg/L), NO_2_^-^-N (mg/L), PO_4_^3-^ -P (mg/L), Fe (mg/L), Cu (mg/L), Cr (mg/L) and Zn (mg/L)).

$WQI=\frac{\sum_{i=1}^{n} \frac{C_{i}}{PL_{i}}}{n}$**Eq. (B.2)**

Where: $Ci$ is the concentration of the *i* contaminant; $PLi$ - maximum permissible level of pollutants (MNS4586: 1998) according to Mongolian Agency for Standard and Metrology (MNS, 1998), $n$ - total number of pollutants.

**Table B.2** Water quality index evaluation list. Given are the numerical value of WQI and the water quality classification, as translated from MNS (1989)

| Water quality index | Water quality classification | Uses and treatment | |
| --- | --- | --- | --- |
| <0.3 | Very clean | | No treatment necessary. Suitable for all kind of water usage |
| 0.3-0.9 | Clean | | For fishery. After treatment, use for drinking and food production |
| 0.91-2.5 | Slightly contaminated | | For livestock, recreation, sport. Unsuitable for drinking and food production |
| 2.51-4.0 | Contaminated | | Use for irrigation and industry after proper treatment |
| 4.01-6.0 | Heavily polluted | | After treatment use for heavy industry without body contact. |
| 6.01-10.0 | Dirty | | Unsuitable for any use. Extensive treatment required |

**Table C.1.** Chemical water quality of samples. Given are sample names, element names, units, limit values according to the Mongolian National Standards MNS 4586:1998 and MNS 4943:2015 and the measurement values. Extra high concentrations are marked in bold, MNS-limit violations with red color. NM indicates not measured samples.

|  | Al | As | B | | Ca^2+^ | | Cl^-^ | | | Co | | | Cr | | Cu | | Fe | | K^+^ | | | Mg^2+^ | | Mn | Mo | |  |  |  |
| --- | --- | --- | --- | --- | --- | --- | --- | --- | --- | --- | --- | --- | --- | --- | --- | --- | --- | --- | --- | --- | --- | --- | --- | --- | --- | --- | --- | --- | --- |
| Units | mg/L | µg/L | µg/L | | mg/l | | mg/L | | | µg/L | | | µg/L | | µg/L | | mg/L | | mg/L | | | mg/l | | mg/L | µg/L | |  |  |  |
| MNS | 0.5 | 10 | 500 | | 100 | | 300 | | | 10 | | | 50 | | 10 | | 1 | | 200 | | | 30 | | 0.1 | 250 | |  |  |  |
| B1 | **12.80** | 8.7 | 5 | | 28.4 | | 0.80 | | | 3.9 | | | **15.6** | | **10.5** | | **11.60** | | 3.64 | | | 8.20 | | 0.17 | 2.1 | |  |  |  |
| B3 | 0.26 | 4.9 | 58 | | 46.4 | | 10.50 | | | 0.3 | | | 1.1 | | 2.8 | | 0.25 | | 3.18 | | | 15.70 | | 0.01 | 8.0 | |  |  |  |
| Ba1 | **8.68** | 5.8 | **153** | | 31.4 | | 1.62 | | | 3.1 | | | 11.7 | | **10.9** | | 7.00 | | **5.18** | | | 7.85 | | 0.12 | 4.5 | |  |  |  |
| G1 | 0.15 | **26.0** | 8 | | 33.5 | | 0.80 | | | 0.2 | | | 0.6 | | 1.6 | | 0.20 | | 1.16 | | | 7.09 | | 0.01 | 2.0 | |  |  |  |
| G2 | 1.43 | **14.8** | **99** | | 42.6 | | 7.34 | | | 0.8 | | | 2.8 | | 3.5 | | 1.35 | | 1.63 | | | 14.40 | | 0.04 | 1.9 | |  |  |  |
| G3 | 0.07 | 7.8 | **110** | | 41.4 | | 6.70 | | | 0.1 | | | 0.4 | | 1.5 | | 0.11 | | 1.23 | | | 14.90 | | 0.01 | 2.2 | |  |  |  |
| K1 | 0.29 | 1.6 | 17 | | 19.6 | | 1.93 | | | 0.2 | | | 0.7 | | 2.1 | | 0.31 | | 1.37 | | | 5.11 | | 0.03 | 1.1 | |  |  |  |
| K2 | 2.51 | **12.6** | 66 | | **54.3** | | 10.40 | | | 1.5 | | | 4.3 | | 9.2 | | 2.25 | | 4.30 | | | **19.10** | | 0.05 | **9.6** | |  |  |  |
| K3 | 0.14 | 1.8 | 24 | | 27.4 | | 3.84 | | | 0.1 | | | 0.6 | | 1.9 | | 0.16 | | 1.65 | | | 7.93 | | 0.01 | 2.2 | |  |  |  |
| K4 | 0.32 | 2.1 | 32 | | 31.2 | | 5.44 | | | 0.3 | | | 1.5 | | 3.5 | | 0.33 | | 2.02 | | | 9.05 | | 0.02 | 3.9 | |  |  |  |
| K5 | 0.56 | 2.1 | 29 | | 32.2 | | 5.54 | | | 0.3 | | | 0.9 | | 2.8 | | 0.46 | | 2.13 | | | 9.49 | | 0.03 | 3.5 | |  |  |  |
| K6 | 0.49 | 2.0 | 30 | | 30.9 | | 5.61 | | | 0.3 | | | 1.1 | | 5.3 | | 0.53 | | 2.04 | | | 9.04 | | 0.03 | 3.5 | |  |  |  |
| K7 | 0.37 | 2.0 | 29 | | 30.5 | | 5.45 | | | 0.4 | | | 1.0 | | 2.7 | | 0.42 | | 1.93 | | | 9.06 | | 0.04 | 3.2 | |  |  |  |
| K8 | 0.60 | 2.1 | 30 | | 33.6 | | 6.28 | | | 0.4 | | | 1.1 | | 2.5 | | 0.50 | | 2.26 | | | 9.79 | | 0.03 | 3.5 | |  |  |  |
| K9 | 0.85 | 2.2 | 30 | | 33.7 | | 7.69 | | | 0.6 | | | 1.6 | | 5.3 | | 0.84 | | 2.49 | | | 9.88 | | 0.06 | 3.1 | |  |  |  |
| O1 | 3.73 | 5.2 | 24 | | 24.2 | | 3.41 | | | 1.8 | | | 6.5 | | 8.1 | | 3.04 | | 2.43 | | | 6.13 | | 0.07 | 2.6 | |  |  |  |
| O2 | 3.75 | 4.9 | 18 | | 24.7 | | 3.96 | | | 1.6 | | | 5.3 | | 6.5 | | 2.89 | | 2.54 | | | 6.54 | | 0.09 | 2.5 | |  |  |  |
| O3 | 5.24 | 5.6 | 17 | | 23.9 | | 3.39 | | | 1.7 | | | 5.4 | | 6.0 | | 3.81 | | 2.92 | | | 6.76 | | 0.10 | 2.5 | |  |  |  |
| O4 | 3.74 | 4.4 | 16 | | 24.4 | | 3.31 | | | 1.4 | | | 4.3 | | 5.6 | | 2.91 | | 2.55 | | | 6.59 | | 0.10 | 2.5 | |  |  |  |
| S1 | 0.47 | 1.2 | 9 | | 34.0 | | 2.11 | | | 0.4 | | | 0.9 | | 3.2 | | 0.54 | | 2.15 | | | 7.11 | | 0.03 | **7.2** | |  |  |  |
| S2 | **18.30** | **10.3** | 7 | | 33.9 | | 0.80 | | | **5.3** | | | **21.9** | | **32.1** | | **16.30** | | 4.54 | | | 10.90 | | **0.20** | 2.2 | |  |  |  |
| S3 | 2.91 | 4.2 | 38 | | **54.8** | | **17.30** | | | 1.6 | | | 4.2 | | 6.2 | | 2.67 | | 4.65 | | | **19.60** | | 0.10 | **7.4** | |  |  |  |
| S4 | 1.54 | 3.7 | 25 | | 46.0 | | 4.27 | | | 0.9 | | | 2.6 | | 4.7 | | 1.47 | | 3.47 | | | 13.70 | | 0.05 | 6.7 | |  |  |  |
| Se1 | 0.27 | 1.0 | 28 | | **53.6** | | **16.20** | | | 0.3 | | | 0.8 | | **11.0** | | 0.24 | | 2.04 | | | 9.26 | | 0.01 | 2.2 | |  |  |  |
| Su1 | 0.11 | 0.7 | 4 | | 5.4 | | NM | | | 0.1 | | | 0.5 | | 3.3 | | 0.10 | | 0.68 | | | 0.91 | | 0.01 | 0.9 | |  |  |  |
| T1 | 0.07 | 0.6 | 5 | | 5.9 | | 0.80 | | | 0.0 | | | 0.7 | | 2.3 | | 0.02 | | 0.40 | | | 0.75 | | 0.01 | 0.4 | |  |  |  |
| T2 | 0.12 | 0.6 | **198** | | 7.8 | | 2.30 | | | 0.0 | | | 0.4 | | **11.2** | | 0.04 | | 0.61 | | | 1.09 | | 0.01 | 0.6 | |  |  |  |
| T3 | 0.14 | 1.6 | 14 | | 15.7 | | 8.84 | | | 0.2 | | | 2.2 | | 2.3 | | 0.19 | | 2.75 | | | 2.43 | | 0.11 | 0.8 | |  |  |  |
| T4 | 0.29 | 4.1 | 23 | | 19.3 | | 10.80 | | | 0.3 | | | 1.8 | | 3.1 | | 0.28 | | 2.13 | | | 3.66 | | 0.03 | 2.3 | |  |  |  |
| T5 | 2.49 | 5.1 | 17 | | 20.0 | | 10.40 | | | 1.1 | | | 3.7 | | 6.3 | | 2.06 | | 2.72 | | | 4.18 | | 0.08 | 1.9 | |  |  |  |
| T6 | 1.78 | 5.3 | 18.7 | | 19.4 | | 10.40 | | | 1.2 | | | 3.1 | | 4.9 | | 1.66 | | 2.32 | | | 4.01 | | 0.07 | 1.7 | |  |  |  |
| T7 | 4.68 | 7.0 | 26 | | 24.2 | | 11.60 | | | 2.5 | | | 8.4 | | 9.0 | | 3.89 | | 3.26 | | | 5.69 | | 0.12 | 2.3 | |  |  |  |
| T8 | 1.63 | 3.9 | 16 | | 20.8 | | 1.72 | | | 0.7 | | | 2.0 | | 4.0 | | 1.31 | | 1.81 | | | 5.62 | | 0.04 | 2.6 | |  |  |  |
| Y1 | 0.59 | 1.4 | 5 | | 10.5 | | 0.80 | | | 0.4 | | | 1.3 | | 3.8 | | 0.63 | | 1.14 | | | 2.38 | | 0.02 | 0.9 | |  |  |  |
| Y2 | 0.35 | 1.5 | 5 | | 11.1 | | 0.80 | | | 0.2 | | | 0.8 | | 2.4 | | 0.40 | | 1.09 | | | 2.61 | | 0.02 | 1.0 | |  |  |  |
| Y3 | 0.22 | 1.6 | 9 | | 10.8 | | 0.80 | | | 0.2 | | | 0.7 | | 3.5 | | 0.24 | | 0.99 | | | 2.43 | | 0.01 | 1.1 | |  |  |  |
|  |  |  |  | |  | |  | | |  | | |  | |  | |  | |  | | |  | |  |  | |  |  |  |
|  |  |  |  | |  | |  | | |  | | |  | |  | |  | |  | | |  | |  |  | |  |  |  |
|  |  |  |  | |  | |  | | |  | | |  | |  | |  | |  | | |  | |  |  | |  |  |  |
|  |  |  |  | |  | |  | | |  | | |  | |  | |  | |  | | |  | |  |  | |  |  |  |
|  |  |  |  | |  | |  | | |  | | |  | |  | |  | |  | | |  | |  |  | |  |  |  |
|  |  |  |  | |  | |  | | |  | | |  | |  | |  | |  | | |  | |  |  | |  |  |  |
|  |  |  |  | |  | |  | | |  | | |  | |  | |  | |  | | |  | |  |  | |  |  |  |
|  |  |  |  | |  | | | |  |  | | |  | |  | |  | |  | | |  | |  | |  |  |  |  |
|  | Na^+^ | NH_4_^+^-N | Ni | | NO_3_^-^-N | | | | NO_2_^-^-N | | Pb | | PO_4_^3-^-P | | SO_4_^2-^ | | Sr | | TNb | | | U | | V | | Zn | | |  |
| Units | mg/L | mg/L | µg/L | | mg/L | | | | mg/L | | µg/L | | mg/L | | mg/L | | µg/L | | mg/L | | | µg/L | | µg/l | | µg/l | | |  |
| MNS | - | 0.5 | | 10 | | 9 | | 0.02 | | | | 10 | | 0.1 | | 100 | | 2000 | | 6 | 50 | | 100 | | | 10 | |  | |
| B1 | 5.71 | 0.010 | **10.5** | | 0.047 | | | | 0.006 | | **9.8** | | 0.283 | | 7.76 | | 149 | | 1.440 | | | 3.1 | | **26.6** | | 29.6 | | |  |
| B3 | **22.40** | 0.010 | 2.0 | | 0.047 | | | | 0.006 | | 2.0 | | 0.050 | | **24.10** | | 431 | | 0.635 | | | 21.0 | | 4.0 | | 15.1 | | |  |
| Ba1 | 7.73 | 0.010 | **9.9** | | 0.047 | | | | 0.006 | | 5.2 | | **0.313** | | 9.80 | | 193 | | 0.374 | | | **16.6** | | **23.8** | | 21.5 | | |  |
| G1 | 4.64 | 0.010 | 0.9 | | 0.047 | | | | 0.006 | | 0.4 | | 0.026 | | 7.19 | | 140 | | 0.888 | | | 2.9 | | 1.7 | | 17.4 | | |  |
| G2 | 8.32 | 0.010 | 2.4 | | 0.407 | | | | 0.006 | | 1.3 | | 0.080 | | 15.70 | | 300 | | 0.861 | | | 4.7 | | 7.0 | | 30.2 | | |  |
| G3 | 8.85 | 0.010 | 1.2 | | 0.047 | | | | 0.006 | | 0.5 | | 0.032 | | 15.90 | | 302 | | 0.953 | | | 3.9 | | 3.1 | | 9.0 | | |  |
| K1 | 5.84 | 0.010 | 1.8 | | 0.047 | | | | 0.006 | | 0.9 | | 0.018 | | 11.90 | | 181 | | 0.561 | | | 1.6 | | 1.3 | | 11.5 | | |  |
| K2 | **26.80** | 0.026 | 5.0 | | 0.085 | | | | 0.006 | | 2.9 | | 0.133 | | **23.30** | | **510** | | 0.395 | | | **21.1** | | 10.7 | | 16.7 | | |  |
| K3 | 10.10 | 0.017 | 1.0 | | 0.047 | | | | 0.006 | | 0.6 | | 0.019 | | 15.50 | | 231 | | 0.351 | | | 4.7 | | 1.3 | | 23.1 | | |  |
| K4 | 14.50 | 0.010 | 2.2 | | 0.047 | | | | 0.006 | | **6.9** | | 0.042 | | 20.00 | | 291 | | 0.237 | | | 9.0 | | 2.4 | | **69.3** | | |  |
| K5 | 15.30 | 0.010 | 1.2 | | 0.047 | | | | 0.006 | | 1.1 | | 0.060 | | 19.60 | | 274 | | 0.518 | | | 8.2 | | 3.2 | | 34.0 | | |  |
| K6 | 14.70 | 0.021 | 1.5 | | 0.047 | | | | 0.006 | | 1.6 | | 0.046 | | 20.30 | | 275 | | 0.672 | | | 8.5 | | 3.4 | | 9.0 | | |  |
| K7 | 14.50 | 0.010 | 1.9 | | 0.047 | | | | 0.006 | | 1.9 | | 0.062 | | 19.60 | | 270 | | 0.443 | | | 8.4 | | 2.9 | | 22.7 | | |  |
| K8 | 16.70 | 0.010 | 1.4 | | 0.047 | | | | 0.006 | | 0.6 | | 0.060 | | 20.60 | | 282 | | 0.566 | | | 8.4 | | 3.5 | | 21.0 | | |  |
| K9 | 17.70 | 0.038 | 1.9 | | 0.225 | | | | 0.006 | | 1.0 | | 0.097 | | 20.40 | | 286 | | 0.378 | | | 8.5 | | 4.4 | | 15.9 | | |  |
| O1 | 9.82 | 0.010 | 5.6 | | 0.109 | | | | 0.006 | | **6.5** | | 0.184 | | 10.40 | | 228 | | 0.345 | | | 2.2 | | 11.4 | | 26.3 | | |  |
| O2 | 11.30 | 0.022 | 4.6 | | 0.176 | | | | 0.006 | | 4.7 | | 0.144 | | 10.70 | | 239 | | 0.875 | | | 2.2 | | 9.9 | | 12.6 | | |  |
| O3 | 10.80 | 0.010 | 5.1 | | 0.298 | | | | 0.006 | | 5.2 | | 0.151 | | 9.81 | | 216 | | 0.864 | | | 2.4 | | 10.6 | | **41.4** | | |  |
| O4 | 10.90 | 0.048 | 4.0 | | 0.155 | | | | 0.006 | | 4.1 | | 0.140 | | 9.97 | | 223 | | 0.679 | | | 2.3 | | 8.8 | | 32.1 | | |  |
| S1 | 9.73 | 0.010 | 1.9 | | 0.047 | | | | 0.006 | | 0.7 | | 0.067 | | 9.52 | | 206 | | 0.739 | | | **19.9** | | 2.8 | | 22.8 | | |  |
| S2 | 7.39 | 0.010 | **15.2** | | 0.047 | | | | 0.006 | | **9.5** | | **0.425** | | 8.64 | | 230 | | 1.280 | | | 4.9 | | **35.6** | | **57.0** | | |  |
| S3 | **39.30** | 0.021 | 4.5 | | 1.100 | | | | 0.006 | | 3.3 | | 0.185 | | **76.40** | | **576** | | 1.470 | | | **14.8** | | 12.4 | | 28.5 | | |  |
| S4 | 19.20 | 0.010 | 3.5 | | 0.047 | | | | 0.006 | | 1.4 | | 0.121 | | 21.60 | | 404 | | 0.669 | | | 6.6 | | 8.9 | | 18.3 | | |  |
| Se1 | 16.60 | 0.010 | 2.0 | | **3.160** | | | | **0.024** | | 1.9 | | 0.024 | | **27.10** | | **785** | | 4.000 | | | 2.9 | | 1.5 | | 9.0 | | |  |
| Su1 | 2.46 | NM | 1.0 | | NM | | | | NM | | 0.3 | | 0.006 | | NM | | 33 | | NM | | | 1.3 | | 0.3 | | 9.9 | | |  |
| T1 | 1.64 | 0.010 | 0.8 | | 0.048 | | | | 0.006 | | 0.3 | | 0.028 | | 3.67 | | 62 | | 0.418 | | | 0.1 | | 0.3 | | 17.8 | | |  |
| T2 | 2.83 | 0.010 | 0.7 | | 0.047 | | | | 0.006 | | 1.3 | | 0.014 | | 13.20 | | 65 | | 0.996 | | | 0.2 | | 0.3 | | 14.4 | | |  |
| T3 | 10.60 | **3.610** | 1.0 | | 0.285 | | | | 0.007 | | 1.1 | | **0.341** | | 8.67 | | 144 | | **4.600** | | | 0.4 | | 0.8 | | 12.5 | | |  |
| T4 | 11.00 | 0.035 | 2.1 | | **1.480** | | | | 0.008 | | **5.6** | | 0.115 | | 13.70 | | 205 | | 1.790 | | | 1.3 | | 3.4 | | 17.0 | | |  |
| T5 | 11.20 | 0.019 | 3.5 | | 0.558 | | | | 0.006 | | 2.1 | | 0.184 | | 11.80 | | 207 | | 1.890 | | | 1.9 | | 9.1 | | 31.0 | | |  |
| T6 | 11.20 | 0.010 | 3.2 | | 0.490 | | | | 0.006 | | 3.6 | | 0.179 | | 12.50 | | 212 | | 1.820 | | | 2.0 | | 9.4 | | 14.3 | | |  |
| T7 | 13.30 | 0.010 | 7.0 | | 0.493 | | | | 0.006 | | **6.6** | | **0.223** | | 16.20 | | 256 | | 1.870 | | | 2.7 | | 16.7 | | 33.1 | | |  |
| T8 | 8.89 | 0.010 | 3.2 | | 0.047 | | | | 0.006 | | 1.6 | | 0.080 | | 9.27 | | 193 | | 1.070 | | | 1.9 | | 5.0 | | **43.2** | | |  |
| Y1 | 3.24 | 0.010 | 1.5 | | 0.047 | | | | 0.006 | | 0.8 | | 0.052 | | 6.74 | | 67 | | 0.425 | | | 0.9 | | 1.8 | | 21.5 | | |  |
| Y2 | 3.64 | 0.010 | 1.3 | | 0.047 | | | | 0.006 | | 0.5 | | 0.022 | | 5.91 | | 75 | | 0.227 | | | 0.9 | | 1.3 | | 13.8 | | |  |
| Y3 | 3.50 | 0.010 | 2.4 | | 0.047 | | | | 0.006 | | 0.7 | | 0.012 | | 5.48 | | 77 | | 0.239 | | | 1.0 | | 1.1 | | 12.2 | | |  |
|  |  |  |  | |  | | | |  | |  | |  | |  | |  | |  | | |  | |  | |  |  |  |  |
|  |  |  |  | |  | | | |  | |  | |  | |  | |  | |  | | |  | |  | |  |  |  |  |
|  |  |  |  | |  | | | |  | |  | |  | |  | |  | |  | | |  | |  | |  |  |  |  |
|  |  |  |  | |  | | | |  | |  | |  | |  | |  | |  | | |  | |  | |  |  |  |  |
|  |  |  |  | |  | | | |  | |  | |  | |  | |  | |  | | |  | |  | |  |  |  |  |

Appendix D - PCA results

Electronic supplement to Environmental Monitoring and Assessment

Mining and urbanization affect river chemical water quality and macroinvertebrate communities in the upper Selenga River Basin, Mongolia.

*Dashdondog Narangarvuu, Tuuguu Enkhdul, Erdenesukh Erdenetsetseg, Enkhbat Enkhrii-Ujin, Khurtsbaatar Irmuunzaya, Gunsmaa Batbayar, Khurelpurev Oyundelger, Rita Sau-Wai Yam, Martin Pfeiffer**
* Department of Biogeography, University of Bayreuth, Germany; [martin.pfeiffer@uni-bayreuth.de](mailto:martin.pfeiffer@uni-bayreuth.de)

**Table D.1** Factor loadings and eigenvalues of the unrotated PCA for all sites. Loadings in bold are higher than 0.700 and thus especially important for the respective factor. % Total Variance represents the explained variance of total variance for each factor. Cumulative variance of several factors is also shown in the last line from left to right.

|  | Factor 1 | Factor 2 | Factor 3 | Factor 4 |
| --- | --- | --- | --- | --- |
| Al | **-0.955** | -0.238 | 0.009 | 0.090 |
| NH_4_^+^-N | 0.063 | -0.108 | 0.250 | **-0.834** |
| As | -0.319 | 0.039 | -0.227 | 0.305 |
| B | 0.005 | 0.150 | -0.229 | 0.197 |
| Ca_2_^+^ | -0.286 | **0.867** | -0.153 | 0.161 |
| Cr | **-0.968** | -0.220 | 0.034 | 0.038 |
| Cl^-^ | -0.012 | **0.788** | 0.457 | -0.162 |
| Co | **-0.981** | -0.139 | 0.014 | 0.053 |
| Cu | **-0.821** | -0.097 | 0.170 | 0.210 |
| Fe | **-0.951** | -0.231 | 0.008 | 0.091 |
| PO_4_^3-^-P | **-0.856** | -0.125 | 0.165 | -0.421 |
| K^+^ | **-0.824** | 0.415 | -0.085 | -0.268 |
| Mg_2_^+^ | -0.311 | **0.803** | -0.359 | 0.101 |
| Mn | **-0.909** | -0.161 | 0.142 | -0.271 |
| Mo | -0.219 | **0.759** | -0.487 | -0.107 |
| Na^+^ | -0.171 | **0.909** | -0.059 | -0.229 |
| Ni | **-0.975** | -0.137 | 0.003 | 0.080 |
| NO_3_^-^-N | 0.019 | 0.473 | **0.830** | 0.215 |
| NO_2_^-^-N | 0.019 | 0.473 | **0.830** | 0.215 |
| Pb | **-0.834** | -0.131 | 0.164 | 0.075 |
| SO_4_^2--^ | -0.091 | **0.838** | 0.057 | -0.081 |
| Sr | -0.150 | **0.912** | 0.224 | 0.139 |
| U | -0.200 | 0.663 | -0.557 | -0.100 |
| V | **-0.982** | -0.085 | -0.028 | 0.059 |
| Zn | -0.497 | -0.122 | -0.111 | 0.098 |
| Eigenvalue | 9.919 | 6.336 | 2.635 | 1.495 |
| % Total variance | 39.7 | 25.3 | 10.5 | 6.0 |
| Cum. % variance | 39.7 | 65.0 | 75.5 | 81.5 |

Appendix E - Macroinvertebrates

Electronic supplement to Environmental Monitoring and Assessment

Mining and urbanization affect river chemical water quality and macroinvertebrate communities in the upper Selenga River Basin, Mongolia.

*Dashdondog Narangarvuu, Tuuguu Enkhdul, Erdenesukh Erdenetsetseg, Enkhbat Enkhrii-Ujin, Khurtsbaatar Irmuunzaya, Gunsmaa Batbayar, Khurelpurev Oyundelger, Rita Sau-Wai Yam, Martin Pfeiffer**
* Department of Biogeography, University of Bayreuth, Germany; [martin.pfeiffer@uni-bayreuth.de](mailto:martin.pfeiffer@uni-bayreuth.de)


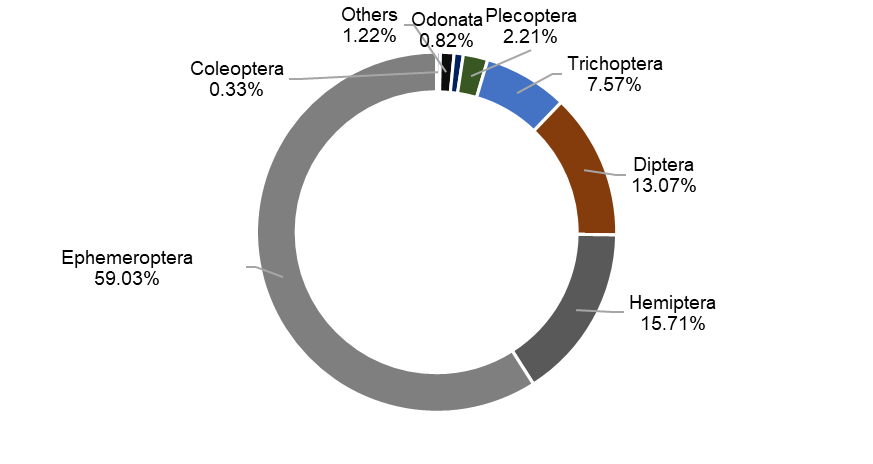


**Fig. E.1.** Relative abundance of macroinvertebrates in all sampling sites.

**Table E.1.** Types and genera of macroinvertebrates found at the survey sites and their tolerance values (Mandaville 2002). Higher values indicate higher tolerance towards organic pollution.

| Taxa name | Abbreviation | Total Abundance | Tolerance value |
| --- | --- | --- | --- |
| **Amphipoda** |  |  |  |
| *Gammarus* | Gam | 41 | 4 |
| **Coleoptera** |  |  |  |
| *Gyretes* | Gyr | 21 | 5 |
| *Lauobius* | Lau | 1 | 4 |
| **Diptera** |  |  |  |
| Chironomi | Chi | 315 | 6 |
| *Limnophila* | Lim | 23 | 3 |
| Orthocladiinae | Ort | 383 | 6 |
| *Pedicia* | Ped | 24 | 6 |
| *Simulium* | Sim | 222 | 7 |
| *Tabanus* | Tab | 3 | 5 |
| **Ephemeroptera** |  |  |  |
| *Acentrella* | Ace | 23 | 4 |
| *Afronurus* | Afr | 1 | 4 |
| *Baetis* | Bae | 2852 | 5 |
| *Brachycentrus* | Bch | 14 | 3 |
| *Caenis* | Cae | 61 | 7 |
| *Cinygma* | Cin | 30 | 2 |
| *Cinygmula* | Ciy | 293 | 4 |
| *Drunella* | Dru | 2 | 0 |
| *Ecdyonurus* | Ecd | 1 | 1 |
| *Epeorus* | Epe | 257 | 0 |
| *Ephemera* | Epa | 37 | 1 |
| *Ephemerella* | Ephe | 39 | 1 |
| *Heptagenia* | Hep | 41 | 4 |
| *Hexagenia* | Hex | 11 | 6 |
| *Isonychia* | Iso | 2 | 2 |
| *Ironodes* c.f. | Iro | 1 | 1 |
| *Leptophlebia* | Lph | 2 | 4 |
| *Nixe* | Nix | 4 | 2 |
| *Oligoneuriella* | Oli | 2 | 2 |
| *Procloeon* | Pro | 661 | 4 |
| *Rhithrogenia* | Rhi | 16 | 0 |
| *Serratella* | Ser | 17 | 2 |
|  |  |  |  |
| **Gastropoda** |  |  |  |
| Gastropoda | Gas | 50 |  |
| **Hemiptera** |  |  |  |
| *Callicorixa* | Cll | 1 | 8 |
| Corixidae | Cor | 1111 | 5 |
| *Saldula* | Sal | 3 | 5 |
| *Sigara* | Sia | 1 | 8 |
| **Odonata** |  |  |  |
| *Gomphus* | Gom | 46 | 5 |
| *Lestes* | Les | 1 | 9 |
| *Stylurus* | Sty | 14 | 4 |
| **Plecoptera** |  |  |  |
| *Agnetina* | Agn | 79 | 2 |
| *Arcynopteryx* | Arc | 62 | 2 |
| *Capnia* | Caa | 4 | 1 |
| *Diura* | Diu | 19 | 2 |
| *Taeniopteryx* | Tae | 1 | 2 |
| **Trichoptera** |  |  |  |
| *Agrypnia* | Agy | 44 | 7 |
| *Anagapetus* | Anp | 3 | 0 |
| *Apatania* | Apa | 83 | 1 |
| *Aphtona becker* | Apt | 3 |  |
| *Arctopsyche* | Ary | 3 | 1 |
| *Brachycercus* | Bra | 26 | 1 |
| *Cheumatopsyche* | Che | 105 | 5 |
| *Geora* | Geo | 27 | 0 |
| *Glossosoma* | Gss | 1 | 1 |
| *Hydrobiosia* | Hyb | 7 | 4 |
| *Hydropsyche* | Hyd | 251 | 4 |
| *Micrasema* | Mir | 7 | 1 |
| *Potamyia* | Pox | 5 | 5 |
| *Psychomiya* | Psy | 10 | 2 |
| *Rhyacophila* | Rya | 2 | 0 |
| **Total** |  | **7368** |  |
|  |  |  |  |

Appendix F - Diversity

Electronic supplement to Environmental Monitoring and Assessment

Mining and urbanization affect river chemical water quality and macroinvertebrate communities in the upper Selenga River Basin, Mongolia.

*Dashdondog Narangarvuu, Tuuguu Enkhdul, Erdenesukh Erdenetsetseg, Enkhbat Enkhrii-Ujin, Khurtsbaatar Irmuunzaya, Gunsmaa Batbayar, Khurelpurev Oyundelger, Rita Sau-Wai Yam, Martin Pfeiffer**
* Department of Biogeography, University of Bayreuth, Germany; [martin.pfeiffer@uni-bayreuth.de](mailto:martin.pfeiffer@uni-bayreuth.de)

**Table F.1.** Diversity pattern of macroinvertebrate communities. Given are taxon number N0, Shannon diversity index (Shannon entropy) H’, Shannon diversity number N1, Simpson diversity number N2, Shannon evenness E10, Simpson Evenness E20, Pileou evenness J.

|  | N0 | H’ | N1 | N2 | E10 | E20 | J |
| --- | --- | --- | --- | --- | --- | --- | --- |
| B1 | 4 | 0.34 | 1.40 | 1.16 | 0.35 | 0.29 | 0.24 |
| B3 | 14 | 1.97 | 7.17 | 5.47 | 0.51 | 0.39 | 0.75 |
| Ba1 | 15 | 1.55 | 4.73 | 3.33 | 0.32 | 0.22 | 0.57 |
| G1 | 16 | 2.03 | 7.64 | 5.02 | 0.48 | 0.31 | 0.73 |
| G2 | 7 | 0.81 | 2.24 | 1.80 | 0.32 | 0.26 | 0.41 |
| G3 | 8 | 1.79 | 5.98 | 4.97 | 0.75 | 0.62 | 0.86 |
| K1 | 14 | 1.98 | 7.26 | 4.91 | 0.52 | 0.35 | 0.75 |
| K2 | 6 | 1.12 | 3.06 | 2.17 | 0.51 | 0.36 | 0.62 |
| K3 | 20 | 1.69 | 5.43 | 3.29 | 0.27 | 0.16 | 0.57 |
| K4 | 14 | 1.90 | 6.71 | 4.67 | 0.48 | 0.33 | 0.72 |
| K5 | 4 | 1.20 | 3.32 | 2.88 | 0.83 | 0.72 | 0.86 |
| K6 | 6 | 0.80 | 2.22 | 1.70 | 0.37 | 0.28 | 0.44 |
| K7 | 11 | 1.03 | 2.81 | 1.78 | 0.26 | 0.16 | 0.43 |
| K8 | 9 | 1.90 | 6.66 | 5.68 | 0.74 | 0.63 | 0.86 |
| K9 | 11 | 1.76 | 5.83 | 4.47 | 0.53 | 0.41 | 0.74 |
| O1 | 4 | 0.68 | 1.98 | 1.59 | 0.49 | 0.40 | 0.49 |
| O2 | 7 | 0.70 | 2.01 | 1.50 | 0.29 | 0.21 | 0.36 |
| O3 | 8 | 1.09 | 2.99 | 1.99 | 0.37 | 0.25 | 0.53 |
| O4 | 6 | 0.82 | 2.27 | 1.87 | 0.38 | 0.31 | 0.46 |
| S1 | 20 | 1.91 | 6.74 | 4.05 | 0.34 | 0.20 | 0.64 |
| S2 | 15 | 2.12 | 8.36 | 6.27 | 0.56 | 0.42 | 0.78 |
| S3 | 7 | 1.61 | 5.00 | 4.15 | 0.71 | 0.59 | 0.83 |
| S4 | 7 | 1.64 | 5.16 | 4.17 | 0.74 | 0.60 | 0.84 |
| Se1 | 4 | 0.96 | 2.60 | 2.32 | 0.65 | 0.58 | 0.69 |
| Su1 | 16 | 2.27 | 9.68 | 7.38 | 0.61 | 0.46 | 0.82 |
| T1 | 15 | 2.28 | 9.76 | 7.26 | 0.65 | 0.48 | 0.84 |
| T2 | 8 | 1.68 | 5.38 | 4.21 | 0.67 | 0.53 | 0.81 |
| T3 | 4 | 0.80 | 2.22 | 1.70 | 0.55 | 0.42 | 0.57 |
| T4 | 9 | 0.52 | 1.68 | 1.30 | 0.19 | 0.14 | 0.24 |
| T5 | 5 | 1.19 | 3.28 | 2.54 | 0.66 | 0.51 | 0.74 |
| T6 | 8 | 1.47 | 4.36 | 3.21 | 0.54 | 0.40 | 0.71 |
| T7 | 4 | 0.52 | 1.69 | 1.32 | 0.42 | 0.33 | 0.38 |
| T8 | 8 | 1.17 | 3.23 | 2.51 | 0.40 | 0.31 | 0.56 |
| Y1 | 12 | 1.78 | 5.93 | 4.09 | 0.49 | 0.34 | 0.72 |
| Y2 | 14 | 1.61 | 5.01 | 3.04 | 0.36 | 0.22 | 0.61 |
| Y3 | 11 | 1.24 | 3.46 | 2.12 | 0.31 | 0.19 | 0.52 |
| **ALL SITES** | **59** | **2.35** | **10.54** | **5.24** | **0.179** | **0.09** | **0.58** |
|  |  |  |  |  |  |  |  |


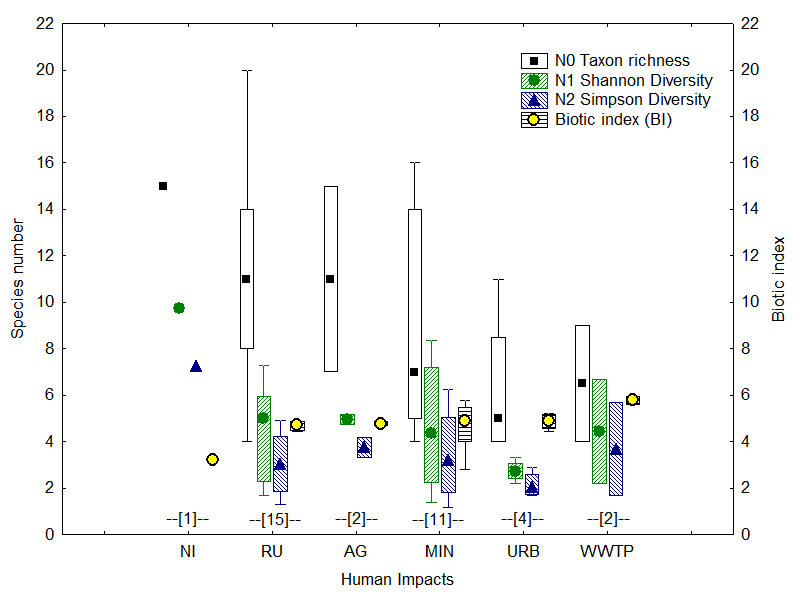


**Fig. F.1.** Macroinvertebrate diversity at the sampling sites demonstrated by effective species numbers (left y-axis) and the respective biotic index (right y-axis). Medians of taxon richness (N0), Shannon diversity (N1) and Simpson diversity (N2) and Biotic index (BI) are grouped according to impact category (NI = no impact, RU = rural, AG = agriculture, MIN = mining, URB = urbanization, WWTP = wastewater treatment plant). Due to the large range of measurements, neither of the measures provided significant differences in statistical testing (Kruskal-Wallis test, n.s.), although medians of several groups differed largely. Number of samples for each group is listed as numbers in square brackets above the X-axis.

Appendix G - Regression analysis

Electronic supplement to Environmental Monitoring and Assessment

Mining and urbanization affect river chemical water quality and macroinvertebrate communities in the upper Selenga River Basin, Mongolia.

*Dashdondog Narangarvuu, Tuuguu Enkhdul, Erdenesukh Erdenetsetseg, Enkhbat Enkhrii-Ujin, Khurtsbaatar Irmuunzaya, Gunsmaa Batbayar, Khurelpurev Oyundelger, Rita Sau-Wai Yam, Martin Pfeiffer**
* Department of Biogeography, University of Bayreuth, Germany; [martin.pfeiffer@uni-bayreuth.de](mailto:martin.pfeiffer@uni-bayreuth.de)

**Table G.1.** Results of multiple regressions of environmental parameters on taxon richness (N0), Shannon Diversity (N1) and Simpson Diversity (N2) of the macroinvertebrate communities at our sampling sites. Given are correlations coefficient R, R2, explained variance adj. R2, F statistics and p values for the multiple regressions as well regression details (standardized and unstandardized regressions coefficients and their standard errors, t-value and p-value) for the respective parameters. All regressions were highly significant.

| 1. Regression Summary for Dependent Variable N0 (Taxon richness), R= 0.79, R²= 0.63, Adjusted R²= 0.56, F_(5.28)_=9.38 p<0.001. | | | | | | | |
| --- | --- | --- | --- | --- | --- | --- | --- |
|  | Beta | Std. Err | B | Std. Err. | t(28) | p-level | Valid N |
| Intercept |  |  | -270.66 | 75.89 | -3.57 | 0.001 |  |
| Longitude | 0.84 | 0.23 | 5.30 | 1.45 | 3.65 | 0.001 | 35 |
| Altitude | 0.83 | 0.23 | 0.02 | 0.00 | 3.64 | 0.001 | 35 |
| DO | 0.32 | 0.12 | 1.10 | 0.42 | 2.61 | 0.014 | 35 |
| EC | -0.74 | 0.16 | -27.90 | 6.05 | -4.61 | 0.000 | 35 |
| U | 0.76 | 0.16 | 0.54 | 0.12 | 4.72 | 0.000 | 35 |
|  |  |  |  |  |  |  |  |
| 1. Regression Summary for Dependent Variable N1 (Shannon Diversity), R= 0.65 R²= 0.42, Adjusted R²= 0.38, F_(2.31)=_11.13 p<0.001. | | | | | | | |
|  | Beta | Std. Err. | B | Std. Err. | t(31) | p-level | Valid N |
| Intercept |  |  | 0.62 | 1.28 | 0.49 | 0.630 |  |
| Altitude | 0.64 | 0.16 | 0.01 | 0.00 | 4.02 | 0.000 | 35 |
| TNb | -0.66 | 0.16 | -1.62 | 0.39 | -4.16 | 0.000 | 34 |
|  |  |  |  |  |  |  |  |
| 1. Regression Summary for Dependent Variable N2 (Simpson Diversity), R= 0.56, R²= 0.31, Adjusted R²= 0.27, F_(2.32)_=7.21 p <0.01. | | | | | | | |
|  | Beta | Std. Err. | B | Std. Err. | t(32) | p-level | Valid N |
| Intercept |  |  | -103.66 | 31.84 | -3.26 | 0.003 |  |
| Longitude | 0.89 | 0.27 | 2.06 | 0.62 | 3.31 | 0.002 | 35 |
| Altitude | 1.01 | 0.27 | 0.01 | 0.00 | 3.79 | 0.001 | 35 |

**Table. G.2** Regression Summary for Dependent Variable: Biotic index (BI). Three parameters Altitude, NH_4_^+^-N and Cl^-^ explain 51% of the variation in the Biotic Index (BI) after exclusion of one outlier. R= 0.74 R²= 0.55 Adjusted R²= 0.51. F(3,30)=12,374 p<0.00002 Std. Error of estimate: =0.49

|  | | | | | | |
| --- | --- | --- | --- | --- | --- | --- |
|  | Beta | Std.Err. | B | Std.Err. | t(30) | p-level |
| Intercept |  |  | 5.19 | 0.34 | 15.23 | 0.000 |
| Altitude | -0,44 | 0.13 | 0.00 | 0.00 | -3.45 | 0.002 |
| Ammonium | 0,29 | 0.13 | 0.33 | 0.15 | 2.26 | 0.031 |
| Cl- | 0,63 | 0.12 | 0.10 | 0.02 | 5.05 | 0.000 |

Appendix H - Biotic index

Electronic supplement to Environmental Monitoring and Assessment

Mining and urbanization affect river chemical water quality and macroinvertebrate communities in the upper Selenga River Basin, Mongolia.

*Dashdondog Narangarvuu, Tuuguu Enkhdul, Erdenesukh Erdenetsetseg, Enkhbat Enkhrii-Ujin, Khurtsbaatar Irmuunzaya, Gunsmaa Batbayar, Khurelpurev Oyundelger, Rita Sau-Wai Yam, Martin Pfeiffer**
* Department of Biogeography, University of Bayreuth, Germany; [martin.pfeiffer@uni-bayreuth.de](mailto:martin.pfeiffer@uni-bayreuth.de)


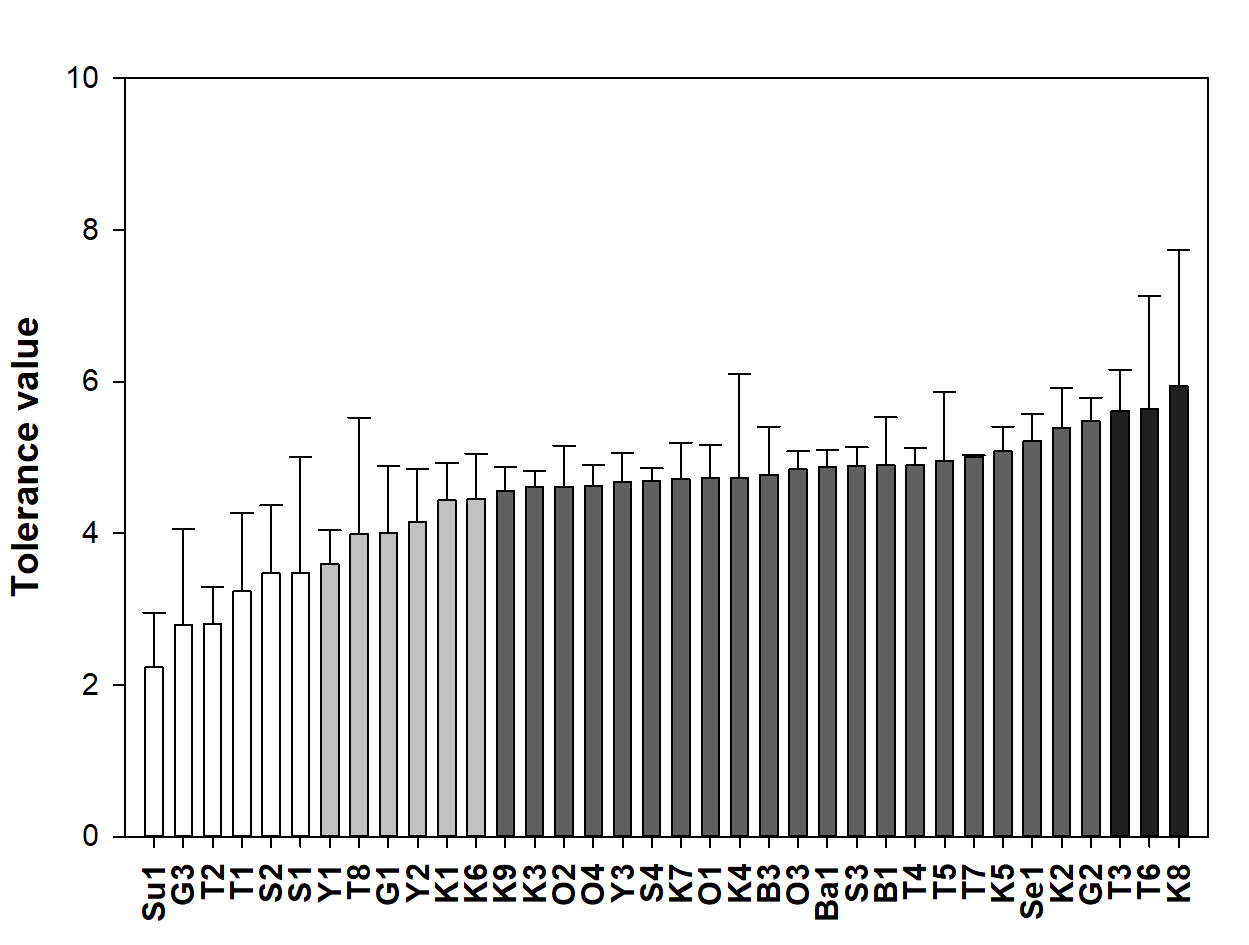


**Fig. H.1.** Distribution of the biotic index along our study sites. The shading refers to the water quality classification, with white = excellent, light gray = very good, grey = good, black = fair. Lower values indicate better water quality, e.g., found in the headwaters of Sugnugur (Su1), Sharyn (S1) and Tuul River (T1, T2). Interestingly, sample sites that were impacted by mining (G3, S2) had excellent water quality according to the biotic index, thus indicating that macroinvertebrates with lower tolerance values could tolerate higher loads of metals. The organisms with the highest tolerance values were found downstream of the WWTPs in Ulaanbaatar (T3) and Darkhan (K8), as well in the mid of Zaamar mining area (T6).
